# *RUNX3* Transcript Variants Have Distinct Roles in Ovarian Carcinoma and Differently Influence Platinum Sensitivity and Angiogenesis

**DOI:** 10.3390/cancers13030476

**Published:** 2021-01-26

**Authors:** Karolin Heinze, Martin Hölzer, Martin Ungelenk, Melanie Gerth, Jürgen Thomale, Regine Heller, Claire R. Morden, Kirk J. McManus, Alexander S. Mosig, Matthias Dürst, Ingo B. Runnebaum, Norman Häfner

**Affiliations:** 1 Department of Gynecology and Reproduction Medicine, Jena University Hospital, 07747 Jena, Germany; kheinze@bccrc.ca (K.H.); matthias.duerst@med.uni-jena.de (M.D.); ingo.runnebaum@med.uni-jena.de (I.B.R.); 2 RNA Bioinformatics and High-Throughput Analysis, Faculty of Mathematics and Computer Science, University of Jena, 07743 Jena, Germany; martin.hoelzer@uni-jena.de; 3 Institute for Human Genetics, Jena University Hospital, 07747 Jena, Germany; Martin.Ungelenk@med.uni-jena.de (M.U.); Melanie.Gerth@med.uni-jena.de (M.G.); 4 Institute for Cell Biology (Cancer Research), Essen University Hospital, 45122 Essen, Germany; juergen.thomale@uni-due.de; 5 Institute for Molecular Cell Biology, Jena University Hospital, 07747 Jena, Germany; REGINE.HELLER@med.uni-jena.de; 6 Research Institute in Oncology and Hematology, University of Manitoba, Winnipeg, MB R3E 0V9, Canada; mordenc1@myumanitoba.ca (C.R.M.); Kirk.McManus@umanitoba.ca (K.J.M.); 7 Department of Biochemistry II, Jena University Hospital, 07747 Jena, Germany; Alexander.Mosig@med.uni-jena.de

**Keywords:** epithelial ovarian cancer, RUNX3, transcript variants, platinum resistance, angiogenesis

## Abstract

**Simple Summary:**

Epithelial ovarian cancer treatment is limited by missing predictive markers, frequent chemotherapy resistance and an incomplete understanding of the biology of tumors. Earlier work proved that hypermethylation of the gene *RUNX3* coding for a transcription factor has prognostic value, and *RUNX3* transcript variant overexpression, regulated by this epigenetic mechanism, influences cisplatin sensitivity and malignant properties of cells contrary. The present data validate *RUNX3* transcript variant-specific effects for high-grade serous ovarian cancer and identify RUNX3-regulated genes and processes. Specifically, DNA damage repair and angiogenesis are influenced by RUNX3, and transcript variant 1 mediates stronger carcinogenic properties.

**Abstract:**

The prognosis of late-stage epithelial ovarian cancer (EOC) patients is affected by chemotherapy response and the malignant potential of the tumor cells. In earlier work, we identified hypermethylation of the *runt-related transcription factor 3* gene (*RUNX3*) as a prognostic biomarker and contrary functions of transcript variants (TV1 and TV2) in A2780 and SKOV3 cells. The aim of the study was to further validate these results and to increase the knowledge about *RUNX3* function in EOC. New *RUNX3* overexpression models of high-grade serous ovarian cancer (HGSOC) were established and analyzed for phenotypic (IC_50_ determination, migration, proliferation and angiogenesis assay, DNA damage analysis) and transcriptomic consequences (NGS) of *RUNX3* TV1 and TV2 overexpression. Platinum sensitivity was affected by a specific transcript variant depending on BRCA background. *RUNX3* TV2 induced an increased sensitivity in BRCA1^wt^ cells (OVCAR3), whereas TV1 increased the sensitivity and induced a G2/M arrest under treatment in *BRCA1*^mut^ cells (A13-2-12). These different phenotypes relate to differences in DNA repair: homologous recombination deficient A13-2-12 cells show less γH2AX foci despite higher levels of Pt-DNA adducts. RNA-Seq analyses prove transcript variant and cell-line-specific *RUNX3* effects. Pathway analyses revealed another clinically important function of RUNX3—regulation of angiogenesis. This was confirmed by thrombospondin1 analyses, HUVEC spheroid sprouting assays and proteomic profiling. Importantly, conditioned media (CM) from *RUNX3* TV1 overexpressing A13-2-12 cells induced an increased HUVEC sprouting. Altogether, the presented data support the hypothesis of different functions of *RUNX3* transcript variants related to the clinically relevant processes—platinum resistance and angiogenesis.

## 1. Introduction

Epithelial ovarian cancer (EOC) is still the most lethal cancer of the female reproductive system, with an average five-year survival rate of 40% [1,2,3,4]. Tumor stage, histology and the (epi-)genetic landscape influence the patient’s outcome. In earlier studies, we showed that *RUNX3* methylation in ovarian carcinoma patients has a prognostic value [5,6] and that RUNX3 protein isoforms function differently in vitro [7]. The aim of the present study was to elucidate the different modes of action—validating and explaining the prior seen contrary functions since very little is known about the difference between the two RUNX3 isoforms. Moreover, analyses should increase the understanding of the relevance of the RUNX3 isoforms for the prognosis of EOC patients.

RUNX3 is one of three members of the runt-related transcription factor family, which are involved in various biological processes [8]. By binding to the cofactor, CBFβ/PEBP2β, it promotes or represses transcription in a tissue-dependent manner [9]. RUNX3 is crucial for T cell differentiation [10] and necessary for the growth of gastric epithelial cells [11]. Interestingly, the chromosomal region 1p36, including the *RUNX3* locus, is a deletion hotspot in different carcinomas [12,13]. The two transcript variants are tightly controlled by two independent promoters. The C-terminus and runt-domain are conserved while a 19 amino acid N-terminal difference discriminates between the two isoforms [13]. RUNX3 is a highly potent transcription factor that is associated with various signaling pathways, including Wnt [8,14], Notch [15,16] and DNA repair [17,18]. By regulating angiogenesis inhibitor thrompospondin-1 (TSP-1) and other angiogenesis regulators [19,20,21,22], RUNX3 influences signaling pathways within a cell and between the cell and its extracellular matrix. Despite studies analyzing pre-invasive [8,23,24] and invasive carcinomas [25,26,27], which were performed in diverse tumor types, the mechanisms by which aberrant RUNX3 expression impacts disease pathogenesis remains poorly understood. Findings in *Runx3* null mice [11] propose a crucial role as a carcinogenic modulator, whereas the discussion on specific roles is highly contradictory. Tumor suppressive [8,27], as well as oncogenic characteristics [28,29], were suggested in different studies performed on different tumor entities. Additionally, RUNX3 exerts both tumor suppressive and oncogenic functions in pancreatic cancer depending on mutational background and phenotypic readout [30]. In the ovary, similar contradicting findings describe RUNX3 as tumor suppressor [31], regulator of normal physiological development [32] or an oncogene [25,33]. In our recent publication, we provided the first evidence that the different roles described for *RUNX3* may additionally depend on the specific transcript variant investigated [7]. Whereas *RUNX3* TV1 overexpression mediated increased platinum resistance and migration RUNX3 TV2 induced the contrary phenotypes. One limitation was the use of the cell-lines A2780 and SKOV3 not representative for the most common EOC subtype high-grade serous ovarian cancer (HGSOC) [34,35,36]. In this study, we present new data using HGSOC cell-lines, confirming our hypothesis of different functions. Additionally, we provide novel insight into the genome-wide regulation of gene expression by RUNX3 variants and their influence on DNA repair and angiogenesis, two clinically relevant pathways.

## 2. Methods

### 2.1. Vector Cloning

For gene transfer, the complementary DNA of *RUNX3* transcript variants 1 (NM_001031680) and 2 (NM_004350) were cloned into pBK-CMV or pCDH by PCR (Supplementary Table S1; [7]).

### 2.2. Cell Culture

A13-2-12, A2780 and OVCAR3 ovarian cancer cells were cultured under standard conditions (37 °C, 5% CO_2_, 90% humidity) in RPMI supplemented with 10% fetal calf serum and 1% pen/strep. Transfected cells were kept in cell-line-specific selection RPMI media comprising 0.3 or 0.4 mg/mL of the selection antibiotic G418 for A2780 and A13-2-12, respectively. Cell lines differed in their genetic properties (Supplementary Table S2) and were obtained from Ansgar Brüning (A2780), purchased at ATCC (American Type Culture Collection, LGC Standards, Wesel, Germany) (OVCAR3) or self-established (A13-2-12). The cell-line A13-2-12 was established from a primary ascites culture after several passages following published protocols [37]. After 35 passages, the cell culture underwent cellular crisis resulting in a permanent growing cell-line (in culture up to passage > 100). Cell line authentication was done by STR profiling and revealed the correct identity depicted by 97% and 100% percent match for A2780 and OVCAR3 (ICLAC match criteria worksheet [38]) compared to STR reference profiles (ATCC and [35]). Genetic testing on *BRCA1* and *RB1* mutation status were done on A13-2-12 and A2780 cells in a PCR product-based sequencing approach (Supplementary Table S3). The detected BRCA1 mutation in A13-2-12 was confirmed in the original HGSOC tumor.

### 2.3. Transfection, Single Cell Clone Generation and Transduction

Ovarian cancer cells were transfected, and A2780 single-cell clones were generated according to published protocols [7]. Because A2780 cells expressing *RUNX3* variant 1 or 2 were established at different time points, two different empty vector controls were included in the experiments. Additionally, cells were transduced using *RUNX3* transcript variant-specific pCDH-CMV-MCS-EF1-Puro lentiviral vectors [39].

### 2.4. Functional Analyses In Vitro

Basic functional assays were performed to assess the cell’s ability for colony formation, migration, cellular vitality and proliferation. Furthermore, effects upon cisplatin exposure were examined by IC50 determination (MTT assay) and flow cytometry as described elsewhere [7]. For IC50 determination, a total of 1 × 10^4^ cells were seeded as technical triplicate in a 96-well plate in a total of 200 µL RPMI medium, cultivated for 24 h and exposed for 48 h to different cisplatin concentrations (between 100 and 0.1 µM). Afterward, the cell survival was measured by MTT assay (Promega, Mannheim, Germany) at 570 nm using a microplate reader (SPECTROstar Omega, BMG Labtech, Freiburg, Germany). After background subtraction, relative values were calculated and analyzed via GraphPad 5.0 software using non-linear regression analyses. For cell cycle analysis, cells were treated with 3 µM cisplatin for 48 h, washed twice with PBS, and fixed in 50% ice-cold ethanol at −20 °C for at least 3 h. After washing out the ethanol with double-deionized water, cells were treated with RNase A and stained with propidium iodide (PI, 50 µg/mL). Cells were measured using a Canto II (BD Biosciences, San Jose, CA, USA). Data were analyzed with FlowJo software (FlowJo, Ashland, OR, USA). For cell proliferation assay, a total of 2500 cells were seeded as technical triplicates in a 96-well plate in a total of 100 µL RPMI medium and cultured for 2 (baseline), 24, 48, 72 and 96 h. To assess the cell number, the CellTiter 96^®^ non-radioactive cell proliferation assay (MTT) from Promega was used similar to the IC50 determination. The ability to form colonies was determined after seeding 500 cells in technical triplicates in 6-well plates and cultured in a 2 mL RMPI medium for 10 days. The established colonies were washed with DPBS +/+ and fixed 20 min at 4 °C with ice-cold 70% EtOH. After washing with double-deionized water, the fixed colonies were air-dried and stained with crystal violet solution for 5 min at RT. Acetic acid (10% concentration) was used to extract the crystal violet pigments from the stained colonies, and the amount of crystal violet was then measured at a wavelength of 630 nm. A migration-scratch assay was used to measure cell migration. A total of 2.5 × 10^6^ cells were seeded in a 6-well plate and grown to 80–90% confluence. Three scratches were introduced into the cell monolayer and washed with DPBS to remove cell debris. To measure just the migratory ability, the cells were further cultivated in a serum-free medium. Images were taken at three predefined areas of the scratches at the time points 0, 6, 12 and 24 h. The analysis was then conducted using the open-source software T-Scratch. The mean wound closure at the three predefined areas represented the result for each independent experiment.

### 2.5. Expression Analyses

Gene and protein expression analyses (real-time RT–PCR, immunocytochemistry, Western blot) were conducted using established techniques [5,7]. Gene-specific primers were used (Supplementary Table S4). For Western blot detection, additional antibodies anti-TSP-1 (A6.1 Thermo Scientific, Dreieich, Germany), HRP-conjugated anti-rabbit (Cell signal technology, USA) and an imaging system with a CCD camera (G:BOX Chemi XX6-Syngene, Frederick, MD, USA) were used.

### 2.6. Fluorescence Microscopy-Based Analysis

DNA double-strand break induction following cisplatin treatment was assessed using indirect immunofluorescence and a γH2AX antibody [40]. Platinum-DNA-adduct (Pt-(GpG)) detection was conducted according to a published protocol [41]. Axio Imager Z.2 + Apotome (Zeiss, Jena, Germany) was used for documentation and ImageJ for the image analysis.

### 2.7. Chromosome Instability Assessments

Chromosome instability (CIN) assays were performed as detailed elsewhere [42,43]. Briefly, chromosome enumeration probes (CEPs) recognizing the pericentric regions of chromosomes 8, 11 and 17 were hybridized to asynchronous samples (and controls) according to the manufacturer (Vysis). Low resolution (20×) images were collected as detailed previously [42,43], using an Axio Imager Z2 (Zeiss) microscope equipped with a Plan-Neofluor 20× objective and an Axiocam HR CCD camera. Images were imported into Imaris v7.7.2 (Bitplane), where nuclear areas were quantified. In general, changes in nuclear areas are typically associated with large-scale changes in DNA content (i.e., ploidy) [44] and, therefore, are used as a surrogate marker of CIN [45,46]. Cells were analyzed for changes in CIN score (CS) values [42,43], which are metrics devised to describe both gains and losses of specific chromosomes (i.e., CEP foci). Briefly, individual CS values for each CEP evaluated (CS_8_, CS_11_ and CS_17_) are calculated by subtracting the observed value from the expected value for each individual CEP (2 foci) within a given nucleus, while the CS_C_ (CIN score combined) is determined by summing the absolute values of each individual CS. All nuclear area and CS values were imported into Prism v7 (GraphPad, San Diego, CA, USA), where two-sample Kolmogorov–Smirnov [KS] tests and Mann–Whitney tests comparing cumulative distribution frequencies and rank orders, respectively, were performed (*p*-value < 0.05 is considered statistically significant). Graphs were generated in Prism and exported to Photoshop CS6 (Adobe, San Jose, CA, USA), where figures were assembled.

### 2.8. Assessment Angiogenic Potential

Pro- or anti-angiogenic effects were tested by endothelial spheroid sprouting assay. Twenty-four-hour conditioned media (CM) (RPMI without supplements) from the cells of interest were used to conduct endothelial sprouting experiments according to a published protocol [47]. In short spheroids from human umbilical vein endothelial cells (HUVEC, 3000 cells per spheroid) were generated in 96-well round bottom plates in endothelial cell medium (Medium 199, 17.5% (*v*/*v*) fetal calf serum (FCS), 2.5% (*v*/*v*) human serum, 7.5 μg/mL endothelial mitogen, 7.5 U/mL heparin, 680 μM glutamine, 100 U/mL penicillin, 100 μg/mL streptomycin, 100 μM ascorbic acid, 1% methylcellulose) for 24 h, embedded in a fibrin matrix and incubated for 24 h in control medium (RPMI, 2% FCS; ± 10 ng/mL VEGF) or conditioned medium. Afterward, spheroids were fixed in 4% paraformaldehyde, washed twice with PBS and photographed. Image analysis quantified the number and length of spheroid sprouts. Furthermore, the presence of the most common effectors within the CM was identified using the Proteome Profiler™ human angiogenesis array kit with the supplied protocol (R&D Systems, Minneapolis, MN, USA).

### 2.9. RNA Sequencing and Computational Analysis

Total RNA of *RUNX3* transcript variant 1 (TV1) and 2 (TV2) transfected A13-2-12 and transduced A2780 cells, and native controls were used in biological triplicates to perform whole transcriptome analysis. Sample concentration was determined using a Qubit 2.0 fluorometer (range: 267–546 ng/µL). The overall quality was assessed via Bioanalyzer (Agilent, Waldbronn, Germany). Subsequently, an Illumina TruSeq Stranded Total RNA kit was used to generate two sequencing libraries consisting of 9 samples each. The kit included rRNA depletion (RiboZero Gold rRNA Removal), cDNA generation, index insertion, adapter inclusion and overall purification. The quality of the library was checked after rRNA depletion and after the last purification again using Bioanalyzer. The strand-specific library was then sequenced on an Illumina NextSeq system (high-output, paired-end, read length 151 nt; San Diego, CA, USA)). Overall, 18 samples were sequenced, yielding between 45.3 and 61.9 million paired-end reads per sample.

After demultiplexing, the quality of the resulting FASTQ files was monitored using FastQC (https://www.bioinformatics.babraham.ac.uk/projects/fastqc/) and MultiQC v0.9 [48]. Low-quality bases at the 3′ end were removed by Trimmomatic v0.36 [49] using a sliding-window approach (window size: 5), a minimum quality score of 28, and a minimum read length of 50 nt. The remaining reads with an rRNA origin were removed using SortMeRNA v2.1 [50]. Only one replicate of the A13-2-12 condition showed a higher amount of rRNA reads (8.96%); however, still in an acceptable range. Quality-adjusted and rRNA-cleaned reads were individually mapped to the human reference primary assembly GRCh38 using HISAT v2.0.4 [51] with default parameters and SAMtools v1.3 [52] for sorting and indexing. The annotation of protein- and non-coding human genes were obtained from Ensembl release 93 [53] and used as an input for featureCounts v1.6.3 [54] with the parameters -pBC and -s 2 (the later one to count strand-specific). Only uniquely assigned reads were counted and provided to DESeq2 v1.16.1 [55] for reading count normalization and pairwise identification of differentially expressed genes. Only genes passing an adjusted *p*-value threshold of 0.05 and with a log2-fold change (log2FC) of at least 1 were considered as significant. The ReportingTools v2.16.0 [56] package was used to generate interactive gene expression box plots, and the PCAGO web service [57] was used to investigate the clustering of replicates and conditions. GO term enrichment and visualization of the most significantly regulated pathways were performed with the Piano package v1.16.4 [58] and DAVID online tool [59]. In addition, the WebGestalt online service [60] was used for GSEA and REVIGO [61] for summarizing and visualizing the enriched categories. The Venn diagrams were generated using the online software of bioinformatics & evolutionary genomics lab at Ghent University. Protein–protein-interaction networks were analyzed with String online tool [62]. All raw read data files were deposited at the European Nucleotide Archive under accession ERP124377. RNA-Seq results were validated via qRT–PCR using *GAPDH* and *actin-beta* as housekeeping genes and gene-specific primers for selected genes (Supplementary Table S4).

### 2.10. Statistical Analysis

The mean ± standard deviation is reported unless otherwise indicated. The statistical analyses were done using Microsoft Excel 2010/365 or GraphPad Prism. The Student’s *t*-test was conducted for pairwise comparisons while the one- or two-way ANOVA with Bonferroni post hoc test was applied comparing multiple groups. Statistical significance was defined by *p* < 0.05. A minimum of three biological replicates was done for all functional experiments.

## 3. Results

### 3.1. Cellular Phenotypes Are Differently Affected by RUNX3 Variants in Different EOC Models

Previously, we provided the first *RUNX3* transcript variant-specific overexpression in vitro models in A2780 and SKOV-3 cells and presented data on the difference in cellular function between both variants [7]. Transfection (A13-2-12) and transduction (OVCAR3 and A2780) were used to generate complementary in vitro cellular models to confirm our previous observations. A13-2-12 and OVCAR3 cells were purposefully chosen as the previous models do not accurately represent high-grade serous ovarian carcinoma (HGSOC) [34,35,36]. Both lines are derived from ascites obtained from HGSOC patients and harbor *p53* mutations (A13-12-12: splice site mutation c.782 + 1G > A; OVCAR3: p.Arg248Gln) with A13-2-12 showing an additional *BRCA1* mutation (p.Ala1752Thr). This mutation is reportedly pathogenic [63].

**Figure 1:**
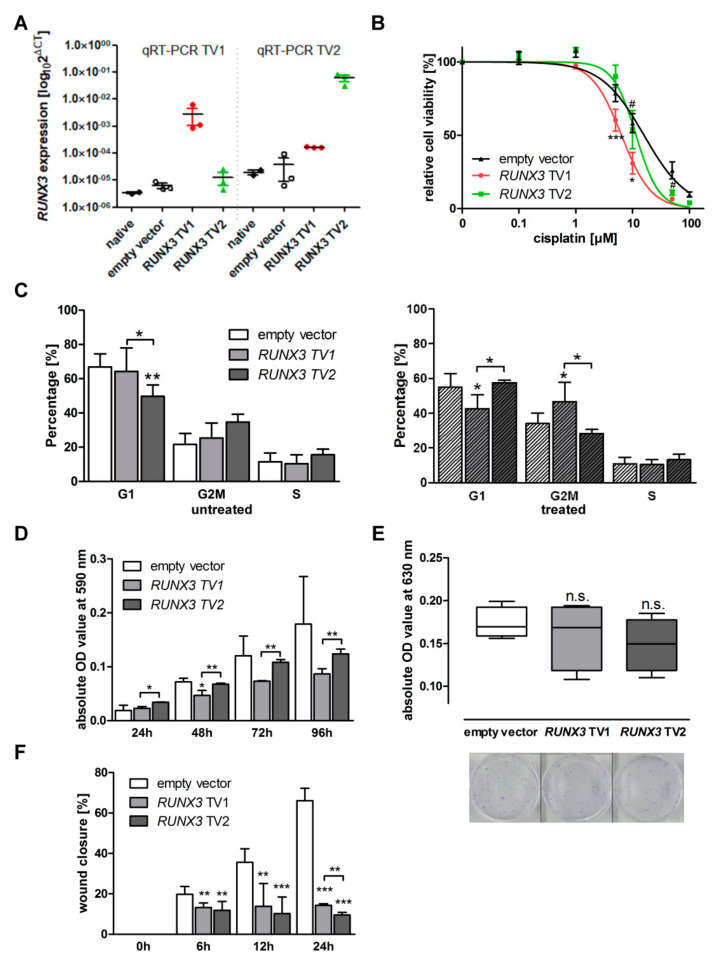
Functional characteristics of *RUNX3* overexpression model of A13-2-12 cells. (**A**) Quantification of *RUNX3* transgene overexpression in A13-2-12 cells compared to the endogenous expression in native and empty vector control cells, *n* = 3. (**B**) IC50 determination against cisplatin. The difference between the variants was significant (using 2-way ANOVA, indicated by #), IC50 values: empty vector 15.9 µM (12.3–20.5 µM), *RUNX3* TV1 6.38 µM (5.48–7.42 µM), *RUNX3* TV2 11.5 µM (9.05–14.6 µM) *n* = 5. (**C**) A13-2-12 cells expressing *RUNX3* TV1 responded with an increase in the proportion of cells in G2-M phase after treatment. *n* = 4. (**D**) The overexpression of TV1 resulted in a reduced proliferation compared to cells expressing TV2. (**E**) *RUNX3* overexpression did not affect the ability to form colonies. Respective crystal violet staining of the colonies is shown below the absolute quantification of the measured optical density (OD) values *n* = 4. (**F**) The migration capacity was significantly reduced by *RUNX3* TV1 and TV2 overexpression compared to empty vector. The strongest effect was present in TV2 expressing A13-2-12 cells compared to the control cells, *n* ≥ 3. (**F**) * *p* < 0.05, ** *p* < 0.01, *** *p* < 0.001 in 2-way ANOVA.

Successful *RUNX3* overexpression was confirmed at mRNA and protein levels (Figure 1A and Supplementary Figure S1A,C). In A13-2-12 cells, *RUNX3* TV1 overexpression led to a significant decrease in cisplatin resistance, whereas TV2 did not change cisplatin sensitivity (IC50_control_ = 15.9 µM, IC50*_RUNX3_*_TV1_ = 6.38 µM; IC50*_RUNX3_*_TV2_ = 11.5 µM; Figure 1B). Cell cycle analysis upon cisplatin exposure confirmed the different phenotype between TV1 and TV2 expressing cells proving an increased G2/M arrest in TV1 cells (Figure 1C, Supplementary Figure S2A).

The migration and proliferation rates were reduced by both transgenes, while distinct differences were observable between variants. Specifically, variant 1 or 2 influenced more strongly proliferation and migration, respectively (Figure 1D,F, Supplementary Figure S2B). The ability to form colonies was not affected by *RUNX3* overexpression in A13-2-12 cells (Figure 1E). In *BRCA1*^wt^ OVCAR3 cells, the expression of *RUNX3* transcript variants had the same effect on the cisplatin resistance as seen in A2780 cells [7]—variant two led to a significant increase in cisplatin sensitivity (Supplementary Figure S1B).

**Figure 2:**
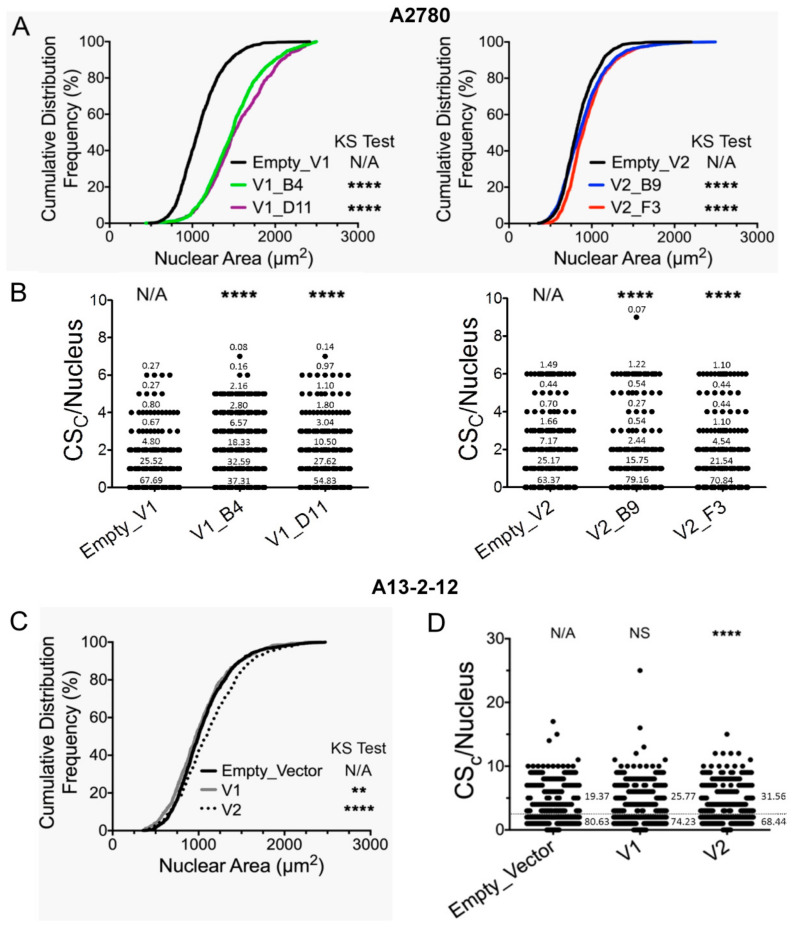
Overexpression of *RUNX3* variants 1 and 2 induces chromosome instability (CIN) in A2780 (**A**,**B**) and A13-2-12 (**C**,**D**). (**A**) Cumulative frequency graphs showing statistically significant differences in the nuclear area distributions of two independent *RUNX3* TV1 (left; V1_B4 and V1_D11) or two TV2 (right; V2_B9 and V2_F3) A2780 clones relative to empty vector controls (KS test; N/A, not applicable; **** *p*-value < 0.0001; *n* > 700 nuclei/condition). (**B**) Scatter plot showing statistically significant differences in CIN score (CS_C_) values for TV1 (left) and TV2 (right) clones relative to empty vector controls (MW test; N/A, not applicable; **** *p*-value < 0.0001; *n* > 700 nuclei/condition). The percentage of cell populations exhibiting specific CS_c_ values are shown within the plots. (**C**) Cumulative frequency graph showing statistically significant differences in nuclear area distributions following TV1 and TV2 overexpression relative to empty vector control in A13-2-12 cells (KS test; N/A, not applicable; ** *p*-value < 0.01; **** *p*-value < 0.0001; *n* > 800 nuclei/condition). (**D**) Scatter plot presenting the overall distribution of CS_c_ values relative to control (MW test; N/A, not applicable; NS, not significant (*p*-value > 0.05]; **** *p*-value < 0.0001; *n* > 800 nuclei/condition). The percentage of cell populations exhibiting specific range of CS_c_ values (CS_c_ ≤ 2 or > 2) are shown within the plots.

Since A13-2-12 and A2780 cells showed contrary phenotypes depending on the overexpressed *RUNX3* variants when treated with cisplatin (e.g., TV1 led to higher sensitivity in A13-2-12 cells but higher resistance in A2780 cells)—further analyses were done with these two cell-lines. Mutation analyses of *RB1* reflected the wildtype sequence that was unchanged after *RUNX3* overexpression and identical between A2780 and A-13-2-12. However, overexpression of both variants was associated with the induction of chromosomal instability (CIN). More specifically, *RUNX3* overexpression in A2780 cells induced a significant increase in the cumulative distribution frequencies of nuclear areas relative to empty vector controls for TV1 (1098 ± 269 µm^2^; 1500 ± 358 µm^2^; 1558 ± 386 µm^2^ nuclear area mean ± SD for control, TV1 clones B4 and D11, respectively; Figure 2A) and to a lesser extent for TV2 (844 ± 217 µm^2^; 898 ± 292 µm^2^; 945 ± 271 µm^2^ nuclear area mean ± SD for control, TV2 clones B9 and F3, respectively; Figure 2A). Both variants led to significant changes in CS_C_ distributions; however, TV1 induced a stronger deviation from the CS_C_ frequency distribution of empty vector controls (Figure 2B), accounting for the larger increase of the nuclear area for TV1 overexpressing A2780 cells. Conceptually, changes in nuclear areas are typically associated with large-scale changes in DNA content (reviewed in [46]), whereas CS_C_ values (see Section 2.7 Chromosome Instability Assessments) are used to describe gains or losses of three specific chromosome enumeration probes (CEP_8_, CEP_11_ and CEP_17_) within a given population of cells [42,43]. Importantly, and in agreement with the heterogeneous nature of CIN, the impact on individual CS values (CS_8_, CS_11_ or CS_17_) was variable (Supplementary Figure S3A). For example, overexpression of TV1 induced significant changes in CS_8_, but not CS_11_, whereas TV2 induced significant changes in CS_8_ and CS_11_, but not CS_17_. These observations are consistent with recent findings showing that CIN can be associated with nonrandom changes (i.e., preferential gains or losses) of specific chromosomes [64,65]. In agreement with the A2780 findings, overexpression of both variants also induced similar, albeit less pronounced changes in A13-2-12 cells (1051 ± 337 µm^2^; 1028 ± 352 µm^2^; 1125 ± 380 µm^2^ nuclear area mean ± SD for control, TV1 and TV2, respectively; Figure 2C). Although only variant 2 overexpression induced a significant change in CS_C_ values, overexpression of both variants induced significant changes in CS_11_ and CS_17_ values, with variant 2 also exhibiting a significant difference for CS_8_ (Figure 2D; Supplementary Figure S3B). Collectively, the above data are consistent with overexpression of both *RUNX3* variants inducing CIN and adversely impacting genome stability. However, data point to a lower CIN induction by the *RUNX3* TV mediating cisplatin sensitivity in the specific cell-line (A2780: TV2; A13-2-12: TV1).

### 3.2. RUNX3 TV1 Increases Cisplatin-Induced DNA Damage in EOC Cells with BRCA^wt^

**Figure 3:**
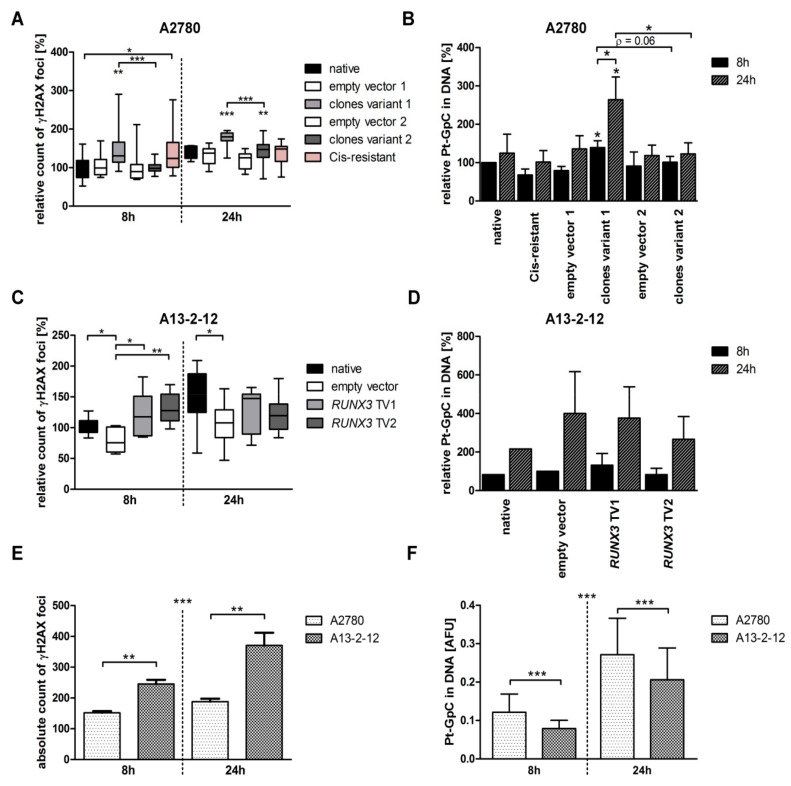
Measurement of cisplatin-induced DNA damage effects. (**A**) Relative γH2AX foci signals per cell in A2780 cells. After 8 h treatment Cis-resistant and *RUNX3* transcript variant 1 overexpressing cells harbored an increase in detected signals compared to parental A2780 or empty vector controls. A significantly higher number of foci were also detected in TV1- compared to TV2-expressing cells. After 24 h, higher numbers of γH2AX foci was also seen in TV2 expressing A2780 cells compared to empty vector cells. However, cells expressing TV1 had still higher DNA damage than TV2 cells at 24 h. *n* ≥ 2, total analyzed cells ≥ 1600. (**B**) Relative level of platinum-DNA-adducts in A2780 cells. Enrichment of the adducts was detected in cells overexpressing *RUNX3* transcript variant 1 in a time-dependent manner. The level of platinum-DNA-adducts differed between cells expressing variant 1 and variant 2, respectively. *n* = 2 (**C**) In A13-2-12 cells, the overexpression of *RUNX3* led in both cases to a higher amount of γH2AX foci after 8 h of cisplatin treatment compared to control cells. This difference disappeared after 24 h. *n* = 2, total analyzed cells ≥ 650. (**D**) A tendency to enrich platinum-DNA-adducts upon longer treatment was seen. No changes showed a statistical significance. *n* = 2. (**E**) Comparing native A2780 and native A13-2-12 cells, A13-2-12 cells showed a higher level of absolute γH2AX foci counts, which further increased over time. (**F**) On the other hand, A2780 cells experienced a stronger accumulation of platinum-DNA-adducts upon cisplatin treatment. * *p* < 0.05, ** *p* < 0.01, *** *p* < 0.001 in Student’s *t*-test.

The different influences on cisplatin sensitivity after *RUNX3* TV1 and TV2 overexpression may be associated with varying levels of DNA damage. Thus, the DNA damage induced by cisplatin was assessed by Pt-DNA adduct and γH2AX foci quantification (Figure 3; Supplementary Figure S4). *RUNX3* TV1 overexpression increased detectable γH2AX foci and the accumulation of DNA adducts in A2780 cells (177% and 264% compared to native A2780 8 h treated, respectively) after cisplatin treatment (5 µM, 24 h). TV2 expressing A2780 cells showed a minor increase of γH2AX foci and DNA adducts by 143% and 123%, respectively. Thus, A2780 cells expressing *RUNX3* TV1 exhibited significantly more DNA damage than TV2 cells indicating different DNA damage repair (Figure 3A,B). In A13-2-12 cells, the transfection itself had a small impact on the accumulation of γH2AX foci (Figure 3C). Transgene expressing cells exhibited increased amounts of γH2AX foci after 8 h, but the comparable level to the empty vector control after 24 h of treatment (5 µM cisplatin, Figure 3C). However, DNA adducts increased after 24 h of cisplatin treatment independent from the specific *RUNX3* variant (Figure 3D). In general, the A13-2-12 cells accumulated more γH2AX foci and fewer DNA adducts than the A2780 cells (mean foci per nuclei after 8 h treatment—A2780:151.9 SD ± 14.3, A13-2-12: 248.5 SD ± 36.0; mean adduct level—A2780:0.12 AFU SD ± 0.05, A13-2-12:0.08 AFU SD ± 0.02). This trend increased over time (Figure 3E,F). On average, this resulted in more γH2AX foci per arbitrary Pt adduct amount in A13-2-12 compared to A2780 cells (after 24 h: 696 γH2AX foci/AFU Pt adduct A2780; 1762 γH2AX foci/AFU Pt adduct A13-2-12). 

Expression analysis of selected genes involved in cell cycle or apoptosis regulation identified different cellular responses to cisplatin between A2780 and A13-2-12. A decline in *p27, E2F1* and *BRCA1* level was seen in exposed A2780 cells, while in A13-2-12 cells, *E2F1* and *BRCA1* experienced an increase in mRNA level (Supplementary Figure S5A). *Survivin* (*BIRC5*) expression was contrary regulated by *RUNX3* variants in both cell-lines under treatment (Supplementary Figure S5B). Whereas *Survivin* showed a reduced expression in *RUNX3* TV1 expressing A13-2-12 cells, this effect was seen in TV2 expressing A2780 cells. The other analyzed genes did not show a different regulation by *RUNX3* variants in both cell-lines under cisplatin exposure (data not shown). However, basal *E2F1* levels in untreated cells were reduced by *RUNX3* TV1 in A2780 cells (Supplementary Figure S5C), which may explain the *RUNX3* TV1 effect on DNA repair (see discussion).

### 3.3. RUNX3 Variants Differentially Regulate the Transcriptome of EOC Cell Lines

**Figure 4:**
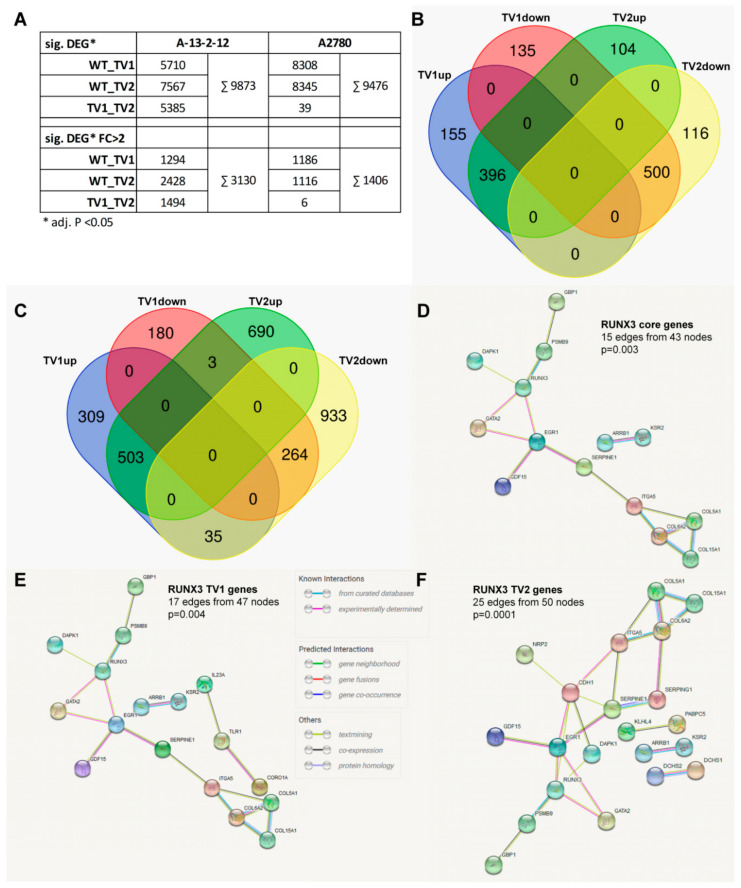
Genome-wide gene expression analysis after *RUNX3* TV1 or TV2 overexpression. (**A**) Overview of the absolute numbers of significant differentially expressed genes (DEGs) (adjusted *p* < 0.05) and the significant DEGs exhibiting a log2FC > 1. (**B**,**C**) Venn-diagram of up- and downregulated DEGs with log2FC > 1 from A2780 (**B**) and A13-2-12 cells (**C**) after *RUNX3* TV overexpression. (**D**–**F**) String protein interaction networks from genes identically regulated by *RUNX3* transcript variants and detected as deregulated in both cell-lines (RUNX3 core genes; (**D**) or from these genes combined with genes specifically regulated by TV1 (**E**) or TV2 (**F**).

To identify regulatory mechanisms, we analyzed the transcriptome of A2780 and A13-2-12 cells after stable overexpression of RUNX3 isoforms. To minimize the sample number and possible variations, transduced A2780 were used instead of the previously used single-cell clones. Through RNA-Seq analyses, 9476 and 9873 significant differentially expressed genes (DEGs) were identified across all pairwise comparisons in A2780 and A13-2-12 cells, respectively (Figure 4A; adjusted *p* < 0.05). Applying a log2foldchange filter (log2FC > 1) 1406 and 3130 DEGs were identified for A2780 and A13-212 (Figure 4A, Supplementary Table S5). Both isoforms regulate a similar number of genes in A2780 (1186 and 1116 genes, representing 1406 unique genes), of which most genes (63.7%; 896/1406) were dysregulated in the same direction (hereafter named RUNX3-regulated genes), and 510 genes were specifically up- or downregulated by one of the isoforms (hereafter named TV-regulated genes; Figure 4B). However, in A-13–2–12 cells, RUNX3 isoforms influence most of the DEGs in an isoform-specific or opposite manner (73.7%; TV-regulated genes 2150/2917; Figure 4C). Identical deregulation was detected for 767 genes (26.3% RUNX3-regulated genes). This difference between the two cell-lines was also detected by the direct comparison of isoform overexpressing cells. Whereas only 6 genes were differentially expressed between A2780 cells overexpressing TV1 or TV2, 1494 genes were detected in A13-2-12 (Figure 4A; Supplementary Figure S6).

The comparison between RUNX3-regulated or TV-regulated genes between both cell-lines revealed a cell-line-specific regulation for the vast majority of genes. From 2622 TV-regulated genes, only 70 genes were differentially expressed in both cell lines, and 97.3% of genes (2552 genes) were cell-line-specific. Similarly, 1571/1617 RUNX3-regulated genes (97.2%) were detected in one cell-line only. From the 46 genes deregulated by RUNX3 in both cell-lines, 30 genes showed an identical change of gene expression, and 16 genes exhibited an opposite regulation in the cell-lines. These 46 genes may represent the core of RUNX3-regulated genes in EOC (Supplementary Table S6). This gene set is enlarged by genes specifically dysregulated by one of the transcript variants (TV1 *n* = 6; TV2 *n* = 7) to define transcript variant-specific gene sets (Supplementary Table S6). Analyses using these gene sets revealed a significantly enriched protein–protein interaction network for RUNX3-deregulated genes (Figure 4D) that is differentially enlarged into transcript variant regulated networks (Figure 4E,F). To identify processes affected by the RUNX3-core or TV-specific genes, we used Gene-Ontology-based analyses. The low number of genes common to both cell-lines precluded a significant enrichment (FDR > 0.05) in specific processes but enabled the identification of Gene-Ontology-based biological processes (GO-BP) containing more than one dysregulated gene (Table 1). RUNX3-dysregulated genes are present in processes related to cell migration, adhesion and angiogenesis. The TV-regulated gene sets add further specific GO-BP, and particularly TV2-regulated genes are detected in 5 additional GO-BP (Table 1). To get a broader picture of the different effects of *RUNX3* TV1 and TV2, we run additional GO analyses using all genes with different expressions between TV1 and TV2 overexpressing cells of A13-2-12 or A2780 (*n* = 1500; 1070 genes included in GO analyses using WebGestalt2019). Table 1 shows the 10 GO-BP with the lowest FDR, proving a different influence of *RUNX3* transcript variants on proliferation, migration and angiogenesis (GO-BP specific genes listed in Supplementary Table S7).

The established data point to a great influence of the cell-line-specific background of common or transcript-specific *RUNX3* functions (see above). Focused GO analyses of angiogenesis, p53 signaling, Wnt signaling, TGF-β signaling, cadherin signaling and NIK/NFκB signaling pathway using all genes significantly affected by *RUNX3* overexpression support this observation (Supplementary Figure S7). However, *RUNX3* overexpression similarly affects most of the analyzed pathways (e.g., inhibiting TGF-β and NIK/NFκB pathway, activating cadherin and wnt signaling) by targeting different genes. The analyses of p53 and angiogenesis signaling revealed a more diverse, cell-line-specific response after *RUNX3* overexpression (Supplementary Figure S7). Because the angiogenesis pathway contained the largest number of deregulated genes (Supplementary Figure S7) and was additionally identified by GO analyses as differently affected by *RUNX3* TV1 vs. TV2 overexpression (Table 1), we focused on further analyses on this pathway.

### 3.4. RUNX3 Variants Differentially Influence Thrombospondin-1 Expression and Angiogenic Effectors

**Figure 5:**
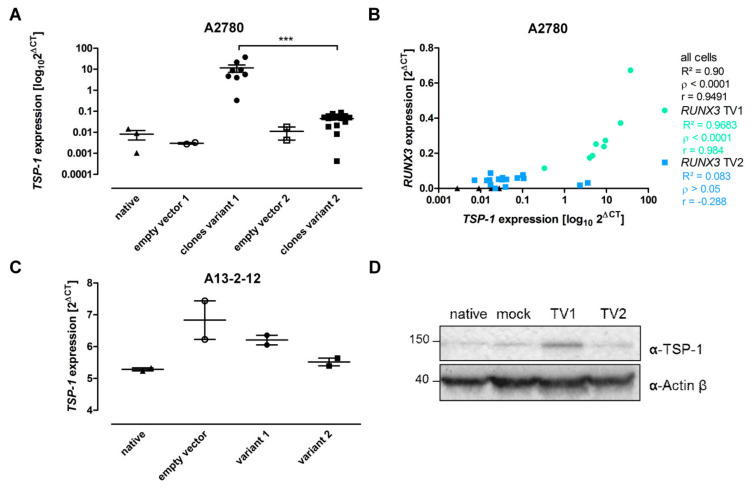
Thrombospondin-1 expression regulation by *RUNX3*. (**A**) *TSP-1* mRNA expression in A2780 cells. *n* ≥ 2, *** *p* < 0.001 in Student’s *t*-test. (**B**) Correlation between RUNX3 expression and *TSP-1* expression in A2780 *RUNX3* single-cell clones. The expression of *RUNX3* TV1 significantly correlated with *TSP-1* expression. *n* (clones) ≥ 8 in Pearson’s correlation. (**C**) *TSP-1* mRNA expression in A13-2-12 cells, *n* ≥ 2. (**D**) Detection of *TSP-1* in A13-2-12 cells by Western blot proved a stronger upregulation of *TSP-1* protein after *RUNX3* TV1 overexpression. Actin-β was used as loading control.

The angiogenesis inhibitor thrombospondin-1 (*TSP-1, THBS1*) is a putative target gene of RUNX3 [22] that was confirmed by mRNA expression level analysis of *TSP-1* in *RUNX3* expressing cells (Figure 5A,C and Supplementary Figure S7). Additionally, *RUNX3* TV1 expressing cells of A13-2-12 and A2780 had higher TSP-1 levels than TV2 expressing cells (Figure 5A,C,D and Supplementary Figure S7). A correlation between *RUNX3* and *TSP-1* mRNA level was seen in A2780 cells (Figure 5B). These results and the genome-wide gene expression analyses indicate a correlation between *RUNX3* expression and *TSP-1* abundance and possible involvement in tumor angiogenesis.

**Figure 6:**
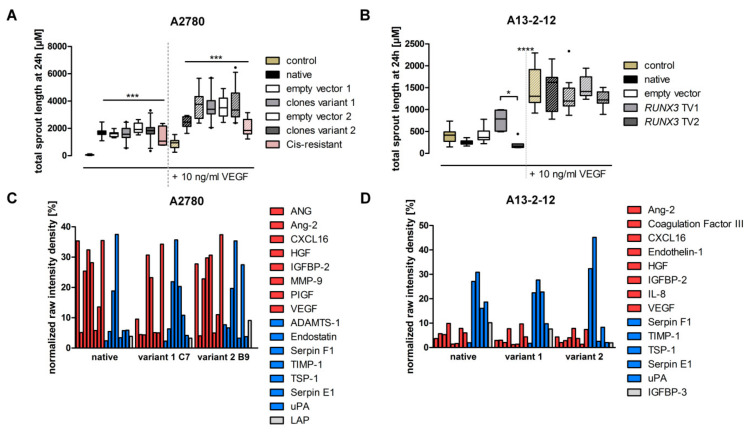
*RUNX3* transcript variant’s effect on the angiogenic effect of EOC cell supernatant. (**A**) Using conditioned media (CM) derived from A2780 cells, considerable amounts of HUVEC sprouting was detected. CM of native A2780 alone led to a 14-fold increase in the amount (Supplementary Figure S5) and a 2-fold increase in sprout length resulting in a 30-fold higher effect compared to RPMI + 2% FSC control. The overexpression *RUNX3* had no effect on the sprouting compared to the native A2780 cells. After the addition of 10 ng/mL vascular endothelial growth factor (VEGF), the CM effect (3-fold increase) was still visible. *n*_spheroid_ ≥ 7. (**B**) The CM from A13-2-12 cells showed a reduced angiogenic potential similar to the control. The addition of 10 ng/mL VEGF led to a significant increase in formed sprouts. While induction of human umbilical vein endothelial cells (HUVEC) sprouting was seen upon incubation with CM from *RUNX3* TV1 expressing A13-2-12 cells, the effect was not significant compared to the empty vector control. The reduction of the sprouting caused by *RUNX3* TV2 CM was also not statistically significant, while the difference between the two variant CM did differ. *n*_spheroid_ ≥ 8. * *p* < 0.05, *** *p* < 0.001, **** *p* < 0.0001 in 2-way ANOVA. (**C**,**D**) Normalized raw intensity data from the Proteome Profiler™ array. Only proteins with signals above the background are shown. Based on current publications, the regulators were either defined as an activator (red), inhibitor (blue) or indifferent (gray). Signals were normalized against the raw intensity of the reference spots on each blot, respectively. Signal quantification of A2780 CM showed more activators than inhibitors, and specifically, high amounts of VEGF were detected (**C**). Quantification of signals from A13-2-12 CM showed higher signal intensities of inhibitory proteins (**D**).

Since the tumor vascularization in vivo is regulated by various factors affecting endothelial cells and our results may indicate RUNX3′s influence on angiogenic modulators, CM from A2780 and A13-2-12 cells was analyzed. HUVEC spheroid experiments showed no difference between the media derived from *RUNX3* TV1 or TV2 expressing A2780 cells (Figure 6A and Supplementary Figure S8). CM from *RUNX3* expressing A13-2-12 cells resulted in different behavior of the HUVEC cells (Figure 6B and Supplementary Figure S8). *RUNX3* TV1 cells increased sprouting in number and length even upon VEGF addition compared to TV2-derived samples (total sprout length 759.2 ± 89.7 vs. 200.2 ± 41.8; Figure 6B). To clarify why A13-2-12 and A2780 cells show different sprouting phenotypes, despite similar effects of RUNX3 on *TSP-1* mRNA level, CM was characterized using the Proteome Profiler™ (Figure 6C,D and Supplementary Figure S9). The increased TSP-1 expression level in *RUNX3* TV1 A2780 and A13-2-12 cells compared to TV2 cells was confirmed. However, several angiogenic activators and a high level of VEGF were detected in CM of A2780, regardless of the transgene status (Figure 6C and Supplementary Figure S9A). Specifically, the high basal VEGF expression in A2780 may mask the angiogenic effects of *RUNX3,* resulting in an unchanged strong positive effect of conditioned media (Figure 6A). Basal CM of A13-2-12 cells had mild effects on HUVEC sprouting, which can be explained by a high portion of angiogenic inhibitors (Figure 6D). For example, native A13-2-12 showed higher levels of TSP-1 than A2780 (Supplementary Figure S9D and Figure 6). The pro-angiogenic effect of CM from *RUNX3* TV1 cells compared to TV2 cells may be caused by the downregulation of pro-angiogenic factors (IL-8 and endothelin) and upregulation of angiogenic inhibitors (SerpinF1 and TIMP-1) by *RUNX3* TV2 (Figure 6D).

## 4. Discussion

RUNX3 is involved in various cellular functions and is described as both tumor suppressor and oncogene. These partial contrary functions may relate to the cellular background [8,27,28,29], functional readout [30] and the specific protein isoform [7] or a combination of these factors. For EOC, the RUNX3 function seems to be clinically relevant as DNA methylation affecting only one of the transcript variants is prognostic for disease progression [5,6]. The aim of the current study was, therefore, the further analysis of functional differences of RUNX3 isoforms, potentially enabling the estimation of their relevance for disease progression. RUNX3 isoforms analyzed in this work originate from full-length transcripts expressed from promoter P1 or P2. Additional transcript variants originating from alternative splicing may exist in vivo, but such variants are not analyzed in this work.

The use of EOC cell-lines not fully representative for HGSOC was one limitation of our previous study [7]. Therefore, we established new overexpression models using the HGSOC cell-lines OVCAR3 and A13-2-12. Both models proved to have a different effect of transcript variants on cisplatin resistance. Whereas OVCAR3, similar to A2780 and SKOV3 cells, showed an increased sensitivity after *RUNX3* TV2 overexpression compared to TV1 expressing cells, the opposite effect was seen in A13-2-12 cells. To verify these results, we additionally performed flow cytometry-based cell cycle analyses for A13-2-12 as published for A2780 [7]. In both cell lines, the sensitivity mediating transcript variant induced a G2/M arrest and a lower level of CIN (A2780 TV2; A13-2-12 TV1). These different results are possibly related to the *BRCA1* mutation in A13-2-12 and its immediate cellular consequences. To elucidate the underlying cause, additional analyses using another *BRCA1* mutated EOC cell line will be necessary. However, further analyses focusing on cisplatin-associated DNA damage, migration, proliferation and gene expression showed a different influence of *RUNX3* variants on the behavior of A2780 and A13-2-12. Whereas more resistant A2780 cells expressing *RUNX3* TV1 showed increased DNA damage (γH2AX and Pt adducts; Figure 3A,B), both *RUNX3* variants had only a small effect on γH2AX foci level, but not on Pt adducts in A13-2-12 after 8 h of treatment (Figure 3C,D). Therefore, *RUNX3* TV1 may directly affect DNA repair mechanisms in A2780, but *RUNX3* variants do not differ in their ability to influence DNA repair in A13-2-12. As E2F1 is involved in and can activate double-strand break (DSB) DNA repair [66], the inhibition of *E2F1* in A2780 *RUNX3* TV1 expressing cells may explain the effect of TV1 (Supplementary Figure S5C). Moreover, disturbed DSB DNA repair in A13-2-12 cells harboring a *BRCA1* mutation may circumvent the *RUNX3* TV1 mediated effect in this cell-line. The DSB DNA repair defect in A13-2-12 may be causative for differences in detected DNA damage levels reflected by Pt adducts and γH2AX foci. A13-2-12 cells show lower levels of adducts after 24 h of treatment, pointing to reduced uptake, increased efflux or higher activity of nucleotide excision repair. Despite the lower adduct levels, the cells acquire a higher level of DSB as marked by γH2AX, likely because of the *BRCA1* mutation and associated DSB repair deficiency. Similarly, higher DNA damage (γH2AX) was detected in intracranial murine models of *BRCA*^mut^ vs. *BRCA*^wt^ breast cancer cell-lines after carboplatin treatment [67] and after inhibition of the Fanconi Anemia-BRCA pathway by *p21* knockout [68]. Besides differences in DNA repair mechanisms, the responsibility of *RUNX3* TV1 or TV2 in mediating cisplatin sensitivity in A13-2-12 or A2780, respectively, may relate to the inhibition of *Survivin* expression by TV1 or TV2. RUNX3 is known to inhibit the promoter of *Survivin* [69], and our data point to a transcript variant-specificity of this effect depending on the cell-line (Supplementary Figure S5).

Although *RUNX3* transcript variants influence cisplatin sensitivity differently depending on the specific cell-line, additional phenotypic properties were differentially impacted in a transcript variant dependent but cell-line independent manner. For example, while cell migration was more strongly inhibited by TV2, proliferation was lower in TV1 expressing cells (Figure 1 and Heinze et al. 2018). To obtain general insights, we analyzed the genome-wide gene expression profile of *RUNX3* TV1 or TV2 overexpressing A2780 and A13-2-12 cells. The acquired data point to strong transcript variant- and cell-line-specific regulation of gene expression (Figure 4); however, Gene Ontology-based analyses revealed regulation of genes coding for hetero- and hemophilic cell adhesion molecules in TV2 overexpressing cells (Table 1). This may explain the stronger influence of this variant on cell migration. Moreover, phosphodiesterase type 5 (PDE5A), described as a mediator of thyroid and breast cancer cell migration [70,71], is specifically upregulated in TV1-overexpressing cells and may be responsible for the increased migration. The general effect of RUNX3 on migration may relate to the presence of genes from migration-associated GO processes within the RUNX3 core genes (Table 1). In relation to the varying proliferation of TV1 vs. TV2 expressing cells, we could not identify a single candidate gene potentially responsible for this effect. Thus, it could be that TV1 or TV2 regulates a number of target genes collectively influencing EOC cell proliferation. Additionally, RUNX3 may affect the proliferation of both cell-lines by targeting different genes. This is confirmed by GO analyses of genes differently expressed between TV1 or TV2-expressing cells of A13-2-12 or A2780 (category II, Table 1), identifying the process of “epithelial cell proliferation”. The largely different gene sets regulated by RUNX3 in both cell-lines (97% of deregulated genes are cell-line-specific) are likely related to the specific cellular background. A2780 does not reflect HGSOC [36], whereas A13-2-12 originates from HGSOC and harbors *p53* and *BRCA1* mutations. The TV-specific target gene regulation in A13-2-12 may additionally explain the known prognostic value of *RUNX3* methylation in HGSOC [5]. TV1, discussed as more oncogenic than TV2, induced the expression of *TIMP3,* whereas TV2 inhibited the expression of this gene in A13-2-12, and high *TIMP3* mRNA expression is associated with reduced progression-free and overall survival of EOC [72].

Besides the use of genome-wide expression data to elucidate phenotypic consequences of RUNX3 overexpression, we aimed to identify processes modified by RUNX3 in EOC cells. Gene ontology-based analyses point to an influence of RUNX3 on angiogenesis (Table 1). We could validate TSP-1 as RUNX3 target gene [22] by NGS and qRT–PCR; however, *RUNX3* TV1 exhibited a pronounced effect compared to TV2 (Figure 5). To estimate the more general effect of both *RUNX3* variants on angiogenesis, we conducted HUVEC spheroid sprouting and proteome profiling assays of conditioned media. CM from A2780 cells did not show an influence of RUNX3 on HUVEC sprouting because of a generally strong pro-angiogenic effect. Proteome profiling revealed high levels of VEGF and other pro-angiogenic factors, potentially masking the increased level of TSP-1 after *RUNX3*-TV1 overexpression (Figure 6). CM from A13-2-12 cells could not induce a similar strong sprouting effect and contained higher levels of angiogenic inhibitors. Surprisingly, albeit *RUNX3*-TV1 induced higher levels of TSP-1, this CM induced an increased HUVEC sprouting pointing to a pro-angiogenic net effect of *RUNX3*-TV1 compared to TV2. This may be caused by the downregulation of pro-angiogenic factors (IL-8 and Endothelin) and upregulation of angiogenic inhibitors (SerpinF1 and TIMP-1) by RUNX3 TV2 (Figure 6D). Altogether, it was shown that *RUNX3* transcript variants do have differential effects on angiogenesis. To fully explain the effects of both *RUNX3* transcript variant on angiogenesis, an expanded pathway analysis should be conducted.

Limitations of our study are (i) the use of overexpression models only, (ii) the missing full elucidation of the mechanisms involved in observed phenotypes and (iii) the analysis of cell culture models only. Therefore, we plan to screen HGSOC cell-lines for their RUNX3 transcript variant expression to identify cell-lines that enable the analysis of knockdown effects. These additional models will then be used to enlighten specific mechanisms of RUNX3 regulation. Moreover, the function of RUNX3 targets identified as potentially involved in phenotype regulation will be validated and can potentially be used to analyze patient material to interrogate the RUNX3 function in vivo.

## 5. Conclusions

**Figure 7:**
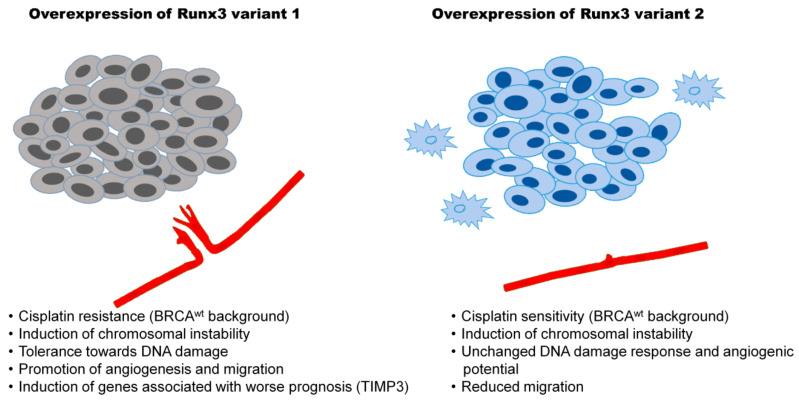
Postulated mode of action of *RUNX3* transcript variants. Whereas present and published data [7] point to TV1 as mediating properties linked to aggressive tumor behavior, TV2 induces phenotypic changes compatible with improved patient survival.

The presented data confirm the hypothesis that *RUNX3* transcript variant 1 and *RUNX3* transcript variant 2 function differently and exert their effects mainly by differential regulation of target gene expression. Albeit known from immune cell development, the different function of transcript variants is rarely considered within other contexts, e.g., cancer development [73,74]. The presented data strengthen the possibility that TV1 has higher oncogenic potential in HGSOC cells, while TV2 presented a more tumor suppressive phenotype (Figure 7). RUNX3 TV1 induces DNA damage tolerance, angiogenesis and migration in vitro. Further investigations in vivo, also in different cancer contexts, must be carried out to fully understand the dichotomy of RUNX3 transcript variants.

## Figures and Tables

**Table 1:** Gene ontology-based analyses of epithelial ovarian cancer (EOC)-related RUNX3 core and transcript variant (TV)-specific pathways.

Gene Category ^a^	Affected Pathways	*n*_genes_	%	*p*-Value	FDR	Genes ^b^
I RUNX3 core (46 genes)	GO:0001764~neuron migration	4	9.09	0.0018	>0.05	GATA2, TUBB2B, NAV1, DDIT4
	GO:0030574~collagen catabolic process	3	6.82	0.0097	>0.05	COL6A2, COL15A1, COL5A1
	GO:0030198~extracellular matrix organization	4	9.09	0.0105	>0.05	ITGA5, SERPINE1, COL6A2, COL5A1
	GO:0001525~angiogenesis	4	9.09	0.0149	>0.05	NRP2, ITGA5, SERPINE1, COL15A1
	GO:0035313~wound healing	2	4.55	0.0207	>0.05	ITGA5, COL5A1
	GO:0007155~cell adhesion	5	11.36	0.0213	>0.05	NRP2, ITGA5, COL6A2, COL15A1, COL5A1
	GO:0031589~cell–substrate adhesion	2	4.55	0.0388	>0.05	CORO1A, ITGA5
I TV1-regulated (core+6 genes)	GO:0007165~signal transduction	8	16.33	0.0238	>0.05	PSD, ARRB1, **TLR1**, **PDE5A**, SRGAP3, COL15A1, GDF15, DAPK1
I TV2-regulated (core+7 genes)	GO:0030198~extracellular matrix organization	5	9.8	0.0018	>0.05	ITGA5, SERPINE1, COL6A2, **CDH1**, COL5A1
	GO:0048565~digestive tract development	3	5.88	0.0039	>0.05	GATA2, FAT4, **DCHS1**
	GO:0007157~heterophilic cell–cell adhesion	3	5.88	0.0079	>0.05	FAT4, ITGA5, **DCHS1**
	GO:0072137~mesenchymal cell proliferation	2	3.92	0.0080	>0.05	FAT4, **DCHS1**
	GO:0007156~homophilic cell adhesion	4	7.84	0.0087	>0.05	DCHS2, FAT4, **CDH1**, **DCHS1**
	GO:0043931~ossification in bone maturation	2	3.92	0.0186	>0.05	FAT4, **DCHS1**
II TV1/2 DEG (1070 genes)	GO:0043062~extracellular structure organization	91	8.50	0	0	CDH1, FN1, PDGFB, PECAM1, TNXB
	GO:0050673~epithelial cell proliferation	73	6.82	2.22 × 10^−15^	9.43 × 10^−13^	CCL2, FGF1, GATA2, NGFR. WNT5A
	GO:0001525~angiogenesis	84	7.85	3.99 × 10^−14^	1.05 × 10^−11^	ANGPT1, EGF, GJA5, THBS2, VEGFC
	GO:0002009~morphogenesis of an epithelium	83	7.76	4.95 × 10^−14^	1.05 × 10^−11^	AREG, DLG3, ESRP2, VDR, WT1
	GO:0001667~ameboidal-type cell migration	71	6.64	8.37 × 10^−14^	1.42 × 10^−11^	ANXA6, DPP4, MEF2C, TACSTD2, TWIST1
	GO:0043588~skin development	73	6.82	3.53 × 10^−13^	5.00 × 10^−11^	BCL2, CDH3, DSC2, KDF1, KRT
	GO:0061564~axon development	81	7.57	1.21 × 10^−12^	1.47 × 10^−10^	APOE,CRABP2, CXCL12, GDNF, NCAM1
	GO:0090130~tissue migration	56	5.23	3.12 × 10^−12^	3.31 × 10^−10^	ADAMTS9, CDH13, SEMA5A, SLIT2, ZEB2
	GO:0001655~urogenital system development	60	5.61	1.27 × 10^−11^	1.16 × 10^−9^	ACE, AR, ESR1, FOXB1, IRX3
	GO:0034330~cell junction organization	55	5.14	1.37 × 10^−11^	1.16 × 10^−9^	CADM1, GJA1, JUP, OCLN, PKP1

^a^ Category I: Genes dysregulated in both cell-lines, RUNX3 core genes are affected by both TV, whereas the TV-specific pathways are dysregulated by a specific TV, Category II: All differentially expressed genes (DEG) between cells overexpressing RUNX3 TV1 or TV2 in A2780 or A13-2-12, ^b^ Genes specifically regulated by TV1 or TV2 are marked in bold, For category II only 5 genes of each pathway are named exemplarily (complete lists in Supplementary Table S7).

## Data Availability

Raw sequencing data are available at the European Nucleotide Archive (Accession: ERP124377). Further data generated during and/or analyzed during the current study are available from the corresponding author on reasonable request.

## Supplementary Materials

The following are available online at https://www.mdpi.com/2072-6694/13/3/476/s1, Figure S1: Functional characteristics of RUNX3 overexpression model in OVCAR3 cells. A Quantification of RUNX3 overexpression in OVCAR3 cells compared to transduced control cells. B IC50 value determination for cisplatin. The transcript variant 2 expressing OVCAR3 cells showed a significantly reduced IC50 value while the overexpression of transcript variant 1 led to no changes in the cisplatin sensitivity (using 2-way ANOVA, *** *p* < 0.001), IC50 values: native 3.91 (2.86–5.36), mock 5.35 (3.81–7.51), RUNX3 TV1 3.38 (2.43–4.70), RUNX3 TV2 1.29 (0.82–2.04), *n* = 3. C RUNX3 immunocytochemical detection confirmed overexpression on protein level, Figure S2: Represantative data of FACS analysis (A) and migration assays (B) of A13-2-12 cells. These data correspond to Figure 1C+F, Figure S3: Overexpression of RUNX3 TV1 and TV2 induces CIN in A2780 (A) and A13-2-12 (B). Scatter plots presenting the gains and losses in CS8 (left), CS11 (middle) and CS17 (right) for each nucleus analyzed for RUNX3 TV1 (upper panels) and TV2 (lower panels) in A2780 (A) and A13-2-12 (B). MW tests evaluating signficant differences in rank order of gains and losses relative to empty vector control (N/A, not applicable; NS, not significant [*p*-value > 0.05] * *p*-value < 0.05; ** *p*-value < 0.01; *** *p*-value < 0.001; **** *p*-value < 0.0001; *n* > 700 nuclei/condition), Figure S4: Represantative data of γH2AX foci (A) and Pt-addukt analysis (B) after ciplatin tretament. These data correspond to Figure 3A+B (A2780) and Figure 3C+D (A13-2- 12). Figure S5: Results of targeted expression analysis upon cisplatin treatment. The cell cylce associated genes p27 and E2F1, the DNA double-strand break response gene BRCA1 and the apoptosis regulators cIAP2, XIAP and Survivin were tested. A In native A2780 cells the cisplatin treatment led to a reduction in BRCA1, p27, E2F1 and Survivin. No changes were seen in the mRNA level of XIAP, while the level of cIAP2 increased. Native A13-2-12 cells only responded with an increase in E2F1 and BRCA1 expression level. The basal level in p27, BRCA1 (higher in A2780), XIAP and cIAP2 (higher in A13-2-12) differed in the untreated state between cell lines. The shaded bars respresent the treated samples. B The cisplatin induced changes of Survivin expression relative to the empty vector control cells differed between both the RUNX3 transcript variants and the cell lines. Whereas TV2 overexpression mediates a reduced Survivin expression in A2780 under treatment a similar effect was detected in A13-2-12 cells after TV1 expression. C RUNX3 TV1 reduced the basal expression of E2F1 in A2780. (*n* = 2, * *p* < 0.05, ** *p* < 0.01, *** *p* < 0.001 in student’s *t*-test.) Figure S6: Descriptive data of RNA-Seq analysis. A MA expression dot plots based on the shrinkage of log2fold changes in A2780 and A13-2-12 cells analysis. Significant DEGs were depicted in red. B Comparative tree clustering of the processed read counts per RNASeq sample. Sample annotation: A2780 = “new”, A13-2-12 = “old”. Figure S7: GO analysis for selected signaling pathways. The RNA-Seq data was used to generate pathway specific heatmaps of up- and downregulated genes involved in the respective pathway. Upregulations are shown in red, downregulations in blue. Numbers correspond to log2 fold changes obtained from the corresponding pairwise gene expression comparison (columns). Pathway analysis were done on the A angiogenesis, B p53 signaling, C Wnt signaling, D TGF-β signaling, E Cadherin signaling and F NIK/NFκB signaling pathway, Figure S8: Detailed data on RUNX3 transcript variant’s effect on HUVEC sprouting. A+B Absolute count of observed sprouts formed under CM incubation derived from A2780 cells (A) and A13-2-12 cells (B). C+D Measured mean sprout length formed after 24 h under CM incubation. No alterations were seen using the different CMs from A2780 cells (C). In A13-2-12 cells (D) the transgene expression resulted in either increase or reduced mean sprout length. nspheroid ≥ 7. * *p* < 0.05, ** *p* < 0.01, *** *p* < 0.001 in 2-way ANOVA. E+F Exemplary spheroids incubated with CM from A2780 (E) or controls and A13-2-12 (F). The scale bar represents 400 µm, Figure S9: Immunodetection of known regulators of angiogenesis using the Proteome Profiler™ Array. The highest signals are indicated respectively. A 72 h CM from A2780 i native, ii clone C7 RUNX3 variant 1, iii clone B9 RUNX3 variant 2 cells. Numbered spots: 1 ADAMTS-1, 2 Angiogenin (ANG), 3 Angiopoietin-2 (Ang-2), 4 CXCL16, 5 Endostatin, 6 HGF, 7 IGFβP-2, 8 TGFβ1 (LAP), 9 MMP-9, 10 PIGF, 11 Serpin E1, 12 Serpin F1, 13 TIMP-1, 14 Thrompospondin-1 (TSP-1), 15 uPA, 16 VEGF. B 24h CM from A13-2-12 i native, ii RUNX3 variant 1, iii RUNX3 variant 2 cells. Numbered spots: 1 Ang-2, 2 coagulation factor III, 3 CXCL16, 4 Endothelin-1, 5 HGF, 6 IGFβP-2, 7 IGFβP-3, 8 IL-8, 9 Serpin E1, 10 Serpin F1, 11 TIMP1, 12 TSP-1, 13 uPA, 14 VEGF. C Quantification of CM derived from native A13-2- 12 cells generated from two different CM aliquots in independent experiments. No difference in the regulators could be detected. D Comparison of TSP-1 expression in A2780 and A13-2- 12 cells. * *p* < 0.05, ** *p* < 0.01, *** *p* < 0.001 in student *t*-test, Table S1: Primer information for cloning, Table S2: Cell line information, Table S3: Primer information for mutational analysis of BRCA1, pRb, Table S4: Primer information for qRT-PCR, Table S5: NGS results for A13-2-12 and A2780, Table S6: RUNX3 core genes and TV-specific genes common to both cell lines, Table S7: Complete list of genes from the 10 GO-BP processes with the lowest FDR identified from DEG between RUNX3 TV1 and TV2 overexpressing cells.
