# Supplementary material for: RUNX3 Transcript Variants Have Distinct Roles in Ovarian Carcinoma and Differently Influence Platinum Sensitivity and Angiogenesis
Source: Cancers (Basel). 2021 Jan 26;13(3):476. doi: 10.3390/cancers13030476 (PMC7866085; doi:10.3390/cancers13030476)
Supplement: Supplementary file 1 [file cancers-13-00476-s001.zip › Heinze et al Suppl Figures.pdf]

RUNX3 transcript variants have distinct roles in ovarian carcinoma and differently influence platinum sensitivity and angiogenesis.

Karolin Heinze[1], Martin Hölzer[2], Martin Ungelenk[3], Melanie Gerth[3], Jürgen Thomale[4], Regine Heller[5], Claire R. Morden[6], Kirk J. McManus[6], Alexander Mosig[7], Matthias Dürst[1], Ingo B. Runnebaum[1]* and Norman Häfner[1]*

[1]Department of Gynecology and Reproduction Medicine, Jena University Hospital, Germany

[2]RNA Bioinformatics and High-Throughput Analysis, Faculty of Mathematics and Computer Science, University of Jena, Germany

[3]Institute for Human Genetics, Jena University Hospital, Germany

[4]Institute for Cell Biology (Cancer Research), Essen University Hospital, Germany

[5]Institute for Molecular Cell Biology, Jena University Hospital, Germany

[6]Research Institute in Oncology and Hematology, University of Manitoba, Canada

[7]Department of Biochemistry II, Jena University Hospital, Germany

*equally contributing senior authors

Corresponding author:
Norman Häfner, PhD
Jena University Hospital
Department of Gynecology and Reproductive Medicine
Am Klinikum 1
07747 Jena
Germany
e-mail: norman.haefner@med.uni-jena.de
ORCID: 0000-0002-7012-483X

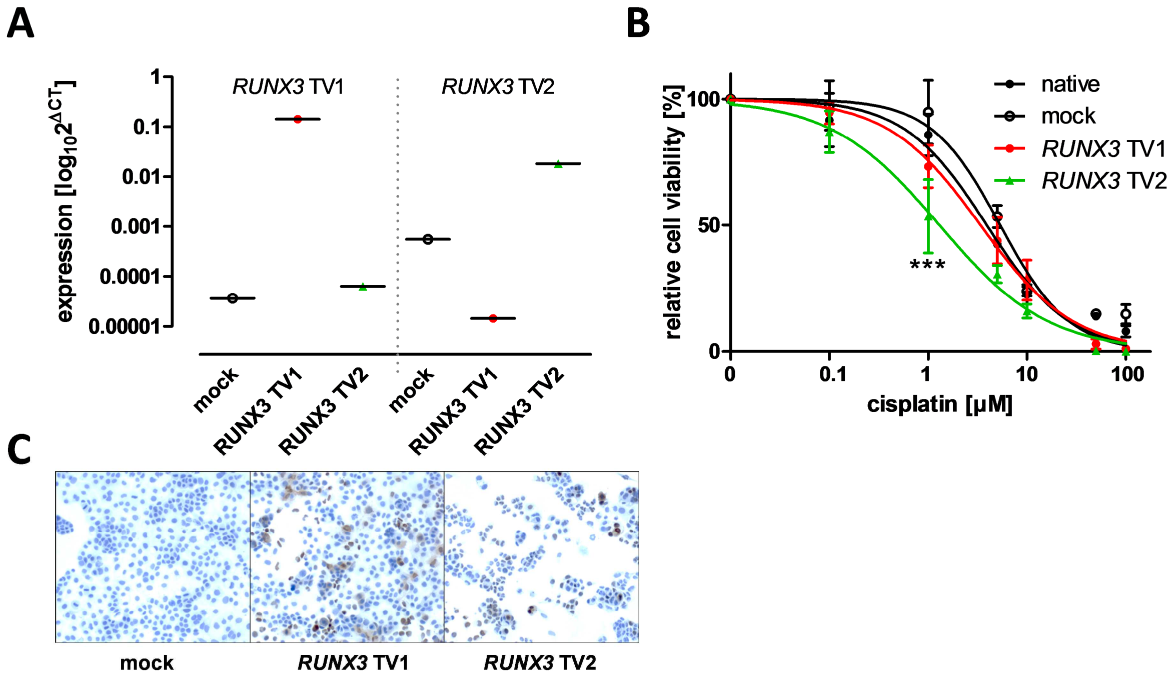

Suppl. Figure S1 Functional characteristics of *RUNX3* overexpression model in OVCAR3 cells. **A** Quantification of *RUNX3* overexpression in OVCAR3 cells compared to transduced control cells. **B** IC50 value determination for cisplatin. The transcript variant 2 expressing OVCAR3 cells showed a significantly reduced IC50 value while the overexpression of transcript variant 1 led to no changes in the cisplatin sensitivity (using 2-way ANOVA, *** $p < 0.001$), IC50 values: native 3.91 (2.86 – 5.36), mock 5.35 (3.81 – 7.51), *RUNX3* TV1 3.38 (2.43 – 4.70), *RUNX3* TV2 1.29 (0.82 – 2.04), n = 3. **C** RUNX3 immunocytochemical detection confirmed overexpression on protein level.

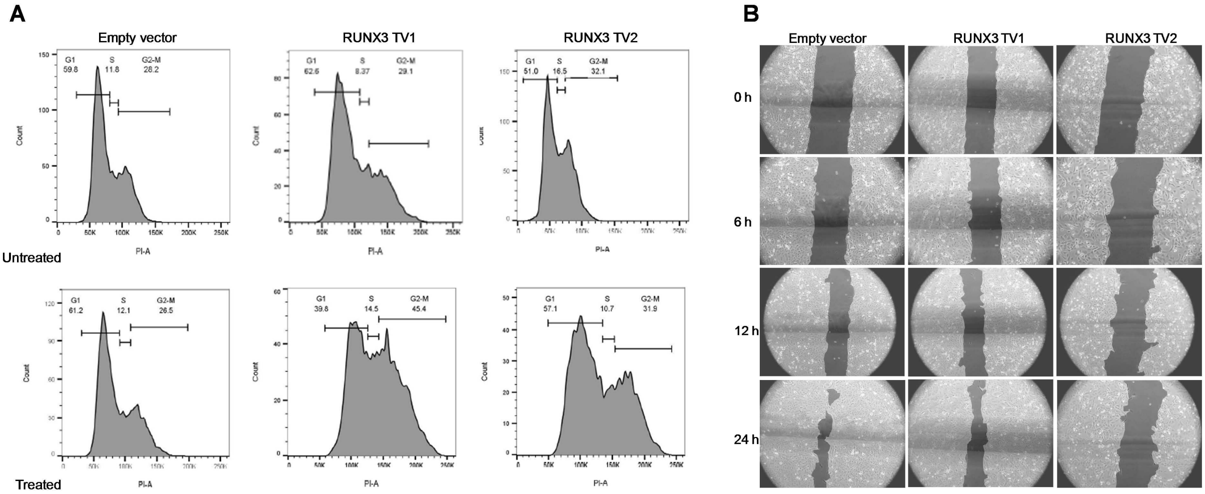

Suppl. Figure S2 Represantative data of FACS analysis (A) and migration assays (B) of A13-2-12 cells. These data correspond to Figure 1C+F.

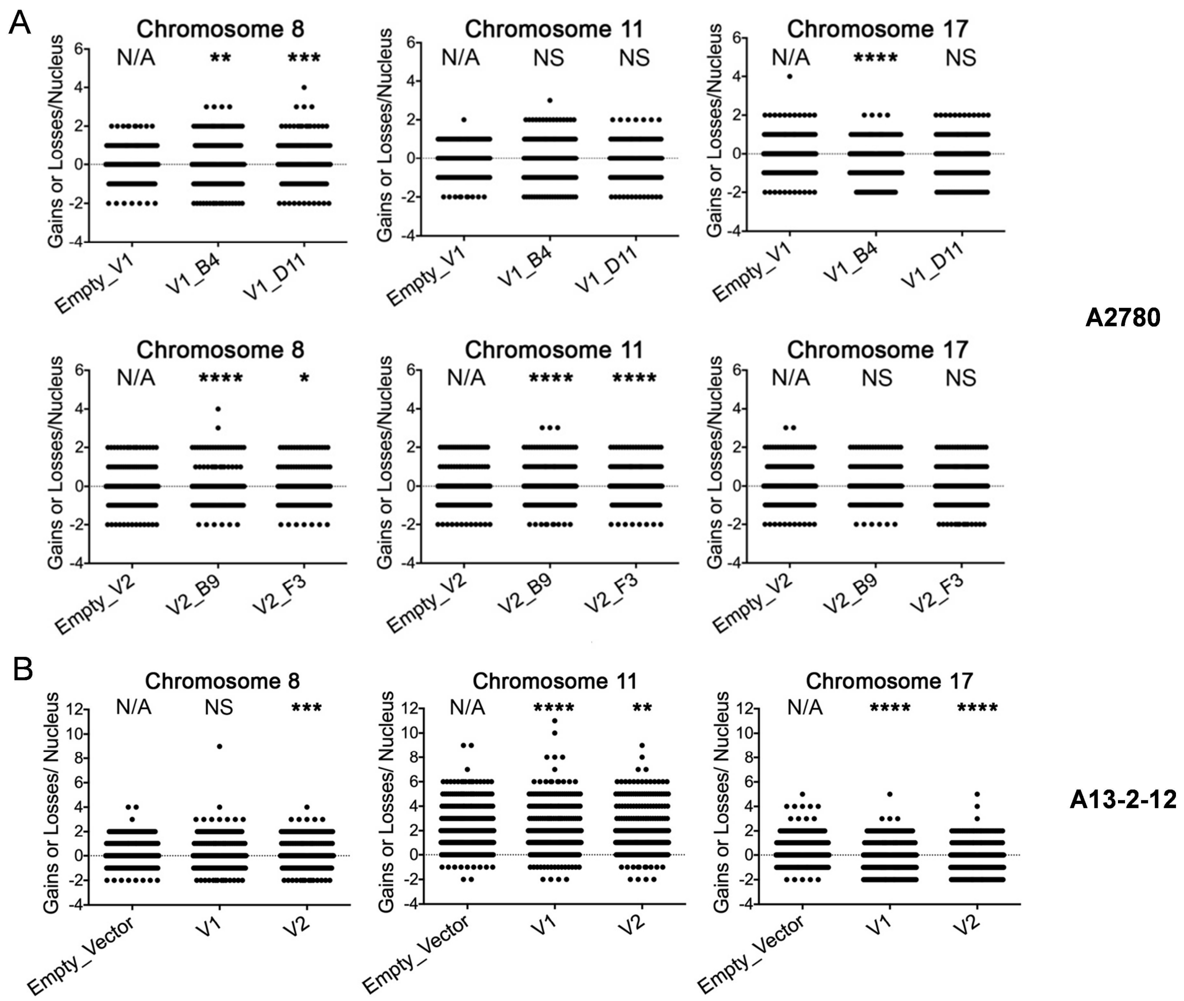

Suppl. Figure S3 Overexpression of *RUNX3* TV1 and TV2 induces CIN in A2780 (**A**) and A13-2-12 (**B**). Scatter plots presenting the gains and losses in CS_8 (left), CS_{11} (middle) and CS_{17} (right) for each nucleus analyzed for *RUNX3* TV1 (upper panels) and TV2 (lower panels) in A2780 (**A**) and A13-2-12 (**B**). MW tests evaluating signficant differences in rank order of gains and losses relative to empty vector control (N/A, not applicable; NS, not significant [p-value > 0.05] * p-value < 0.05; ** p-value < 0.01; *** p-value < 0.001; **** p-value < 0.0001; n > 700 nuclei/condition).

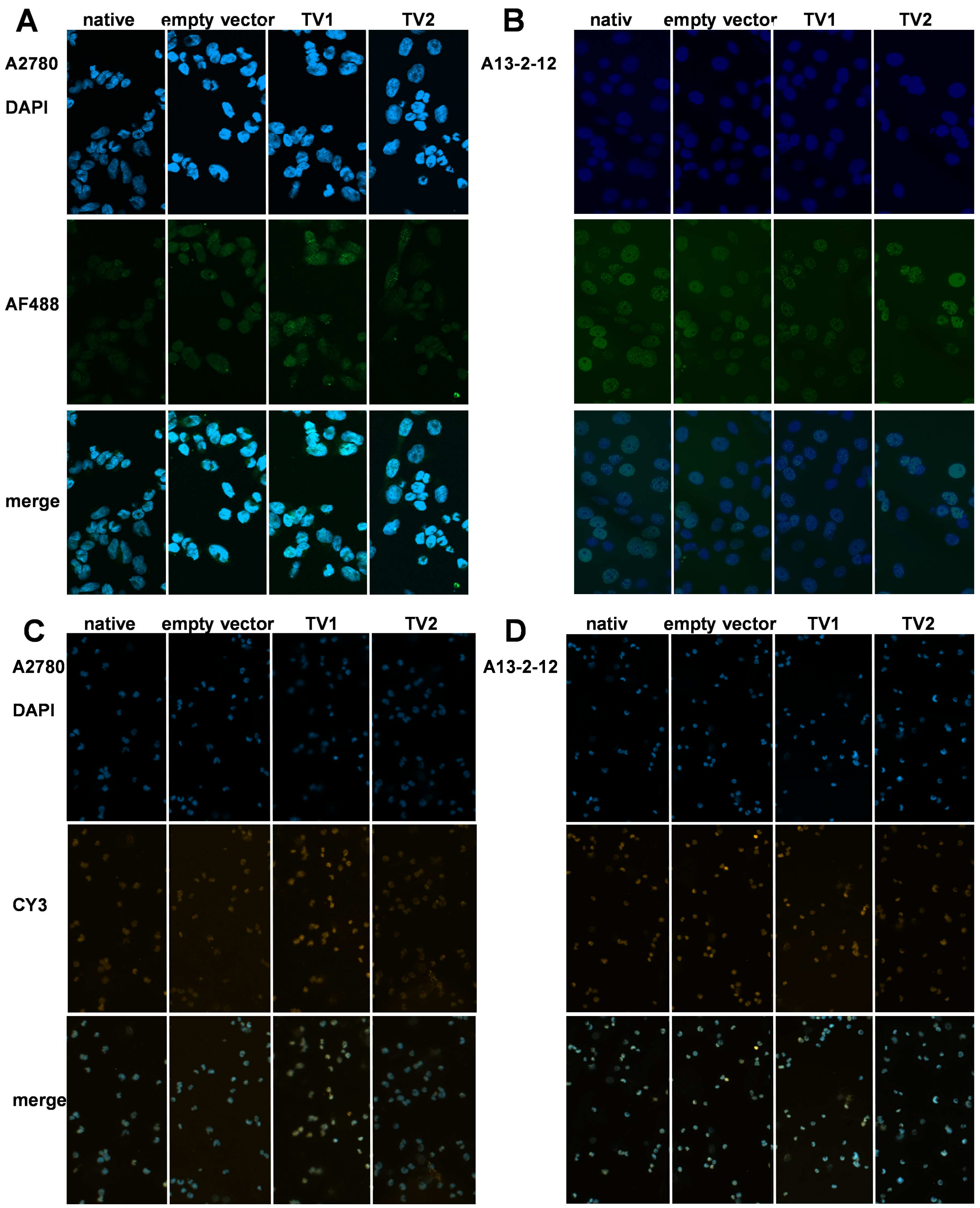

Suppl. Figure S4 Represantative data of γH2AX foci (A) and Pt-addukt analysis (B) after ciplatin tretament. These data correspond to Figure 3A+B (A2780) and Figure 3C+D (A13-2-12).

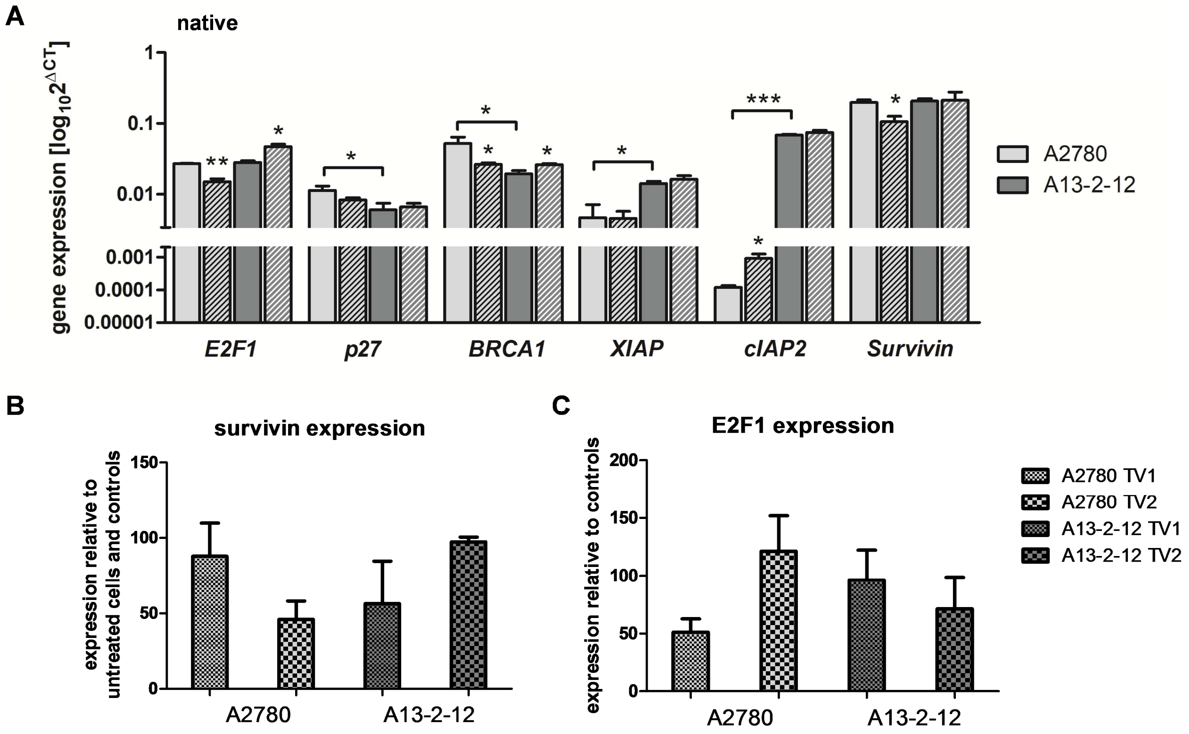

Suppl. Figure S5 Results of targeted expression analysis upon cisplatin treatment. The cell cylce associated genes *p27* and *E2F1*, the DNA double-strand break response gene *BRCA1* and the apoptosis regulators *cIAP2*, *XIAP* and *Survivin* were tested. **A** In native A2780 cells the cisplatin treatment led to a reduction in *BRCA1*, *p27*, *E2F1* and *Survivin*. No changes were seen in the mRNA level of *XIAP*, while the level of *cIAP2* increased. Native A13-2-12 cells only responded with an increase in *E2F1* and *BRCA1* expression level. The basal level in *p27*, *BRCA1* (higher in A2780), *XIAP* and *cIAP2* (higher in A13-2-12) differed in the untreated state between cell lines. The shaded bars respresent the treated samples. **B** The cisplatin induced changes of *Survivin* expression relative to the empty vector control cells differed between both the *RUNX3* transcript variants and the cell lines. Whereas TV2 overexpression mediates a reduced *Survivin* expression in A2780 under treatment a similar effect was detected in A13-2-12 cells after TV1 expression. **C** *RUNX3* TV1 reduced the basal expression of *E2F1* in A2780. (n = 2, * $p < 0.05$, ** $p < 0.01$, *** $p < 0.001$ in student's t-test.)

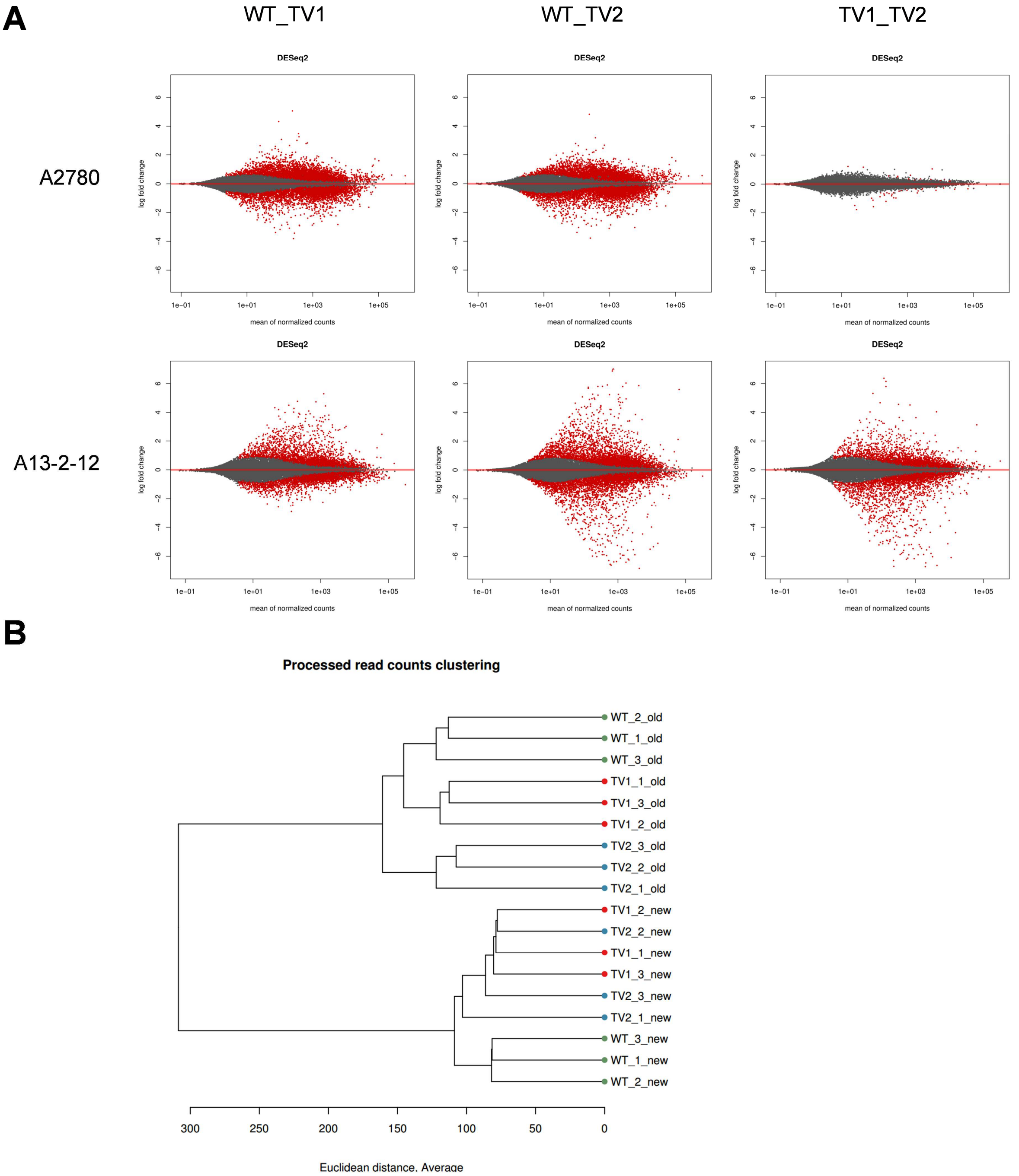

Suppl. Figure S6 Descriptive data of RNA-Seq analysis. **A** MA expression dot plots based on the shrinkage of log2fold changes in A2780 and A13-2-12 cells analysis. Significant DEGs were depicted in red. **B** Comparative tree clustering of the processed read counts per RNA-Seq sample. Sample annotation: A2780 = "new", A13-2-12 = "old".

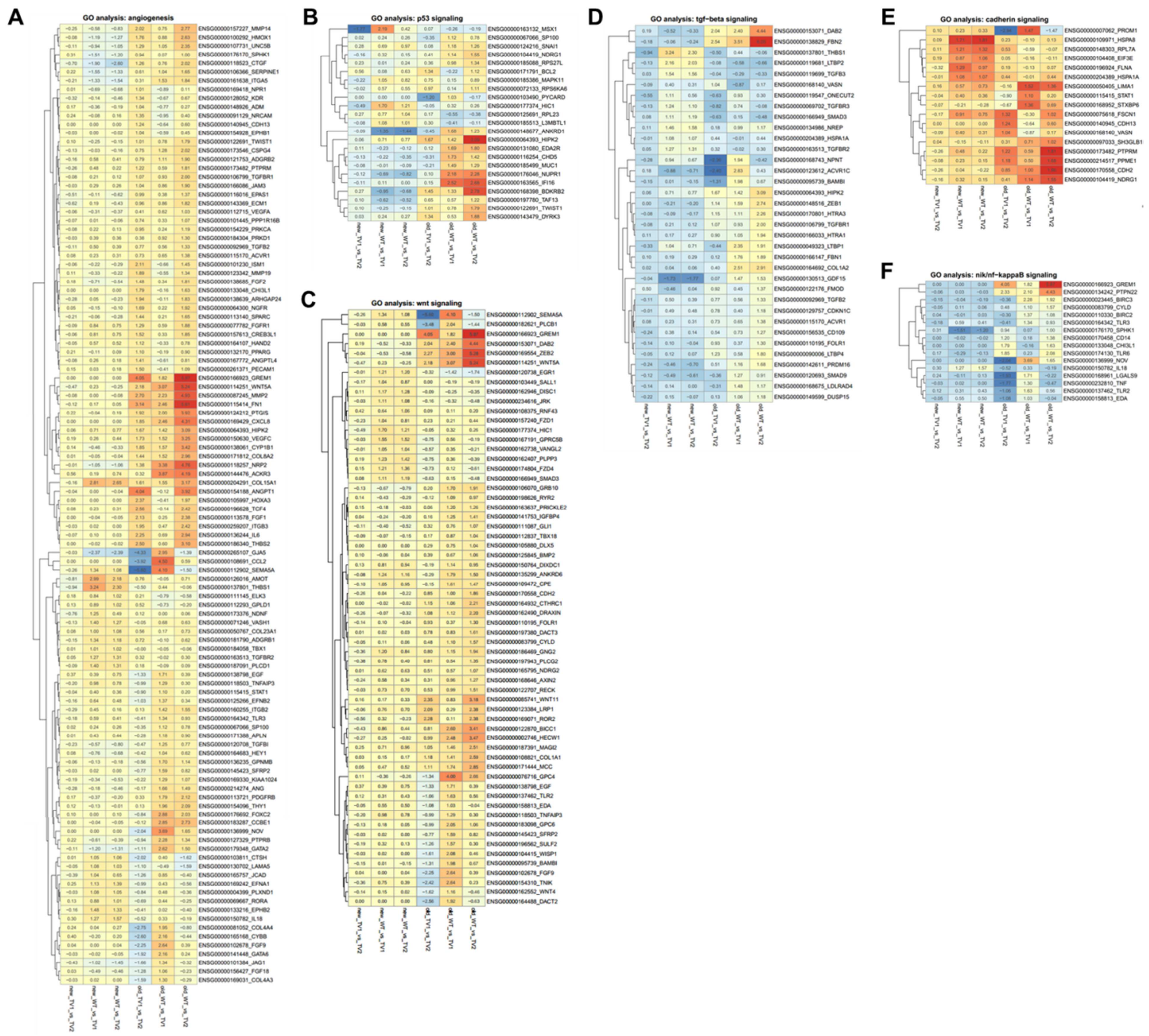

Suppl. Figure S7 GO analysis for selected signaling pathways. The RNA-Seq data was used to generate pathway specific heatmaps of up- and downregulated genes involved in the respective pathway. Upregulations are shown in red, downregulations in blue. Numbers correspond to log2 fold changes obtained from the corresponding pairwise gene expression comparison (columns). Pathway analysis were done on the **A** angiogenesis, **B** p53 signaling, **C** Wnt signaling, **D** TGF-β signaling, **E** Cadherin signaling and **F** NIK/NFκB signaling pathway.

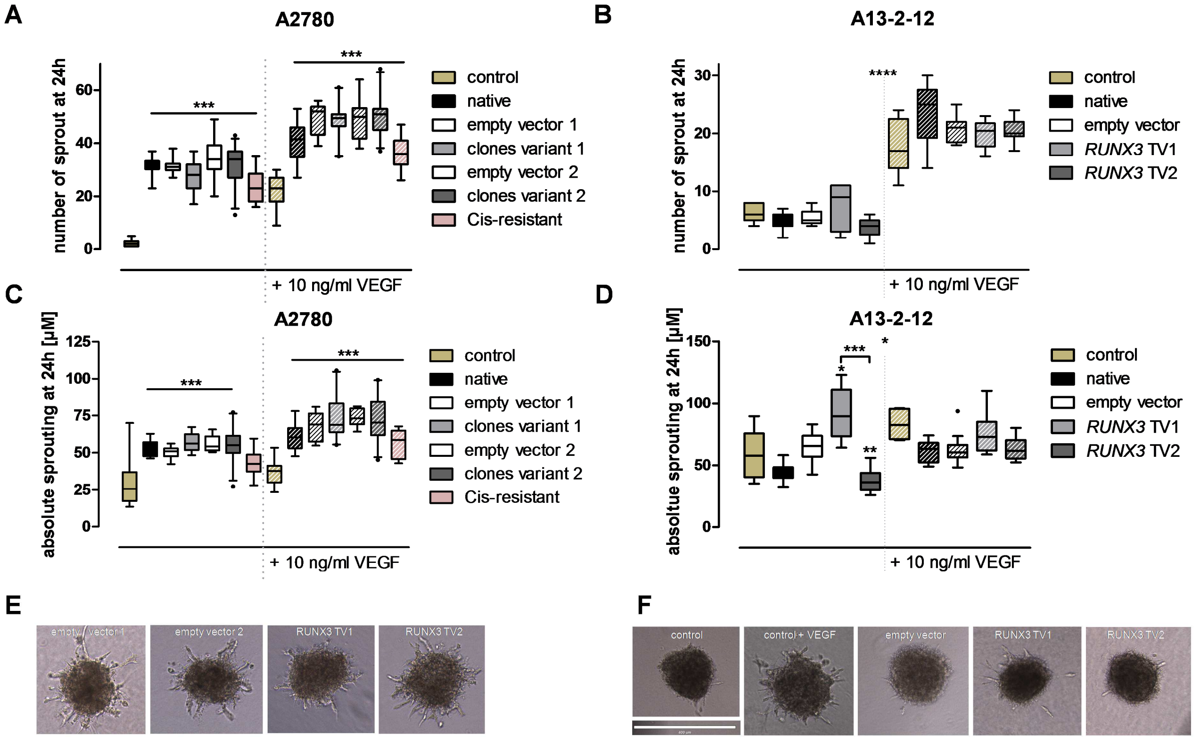

Suppl. Figure S8 Detailed data on *RUNX3* transcript variant's effect on HUVEC sprouting. **A+B** Absolute count of observed sprouts formed under CM incubation derived from A2780 cells (A) and A13-2-12 cells (B). **C+D** Measured mean sprout length formed after 24 h under CM incubation. No alterations were seen using the different CMs from A2780 cells (C). In A13-2-12 cells (D) the transgene expression resulted in either increase or reduced mean sprout length. $n_{spheroid} \geq 7$. * $p < 0.05$, ** $p < 0.01$, *** $p < 0.001$ in 2-way ANOVA. **E+F** Exemplary spheroids incubated with CM from A2780 (E) or controls and A13-2-12 (F). The scale bar represents 400µm.

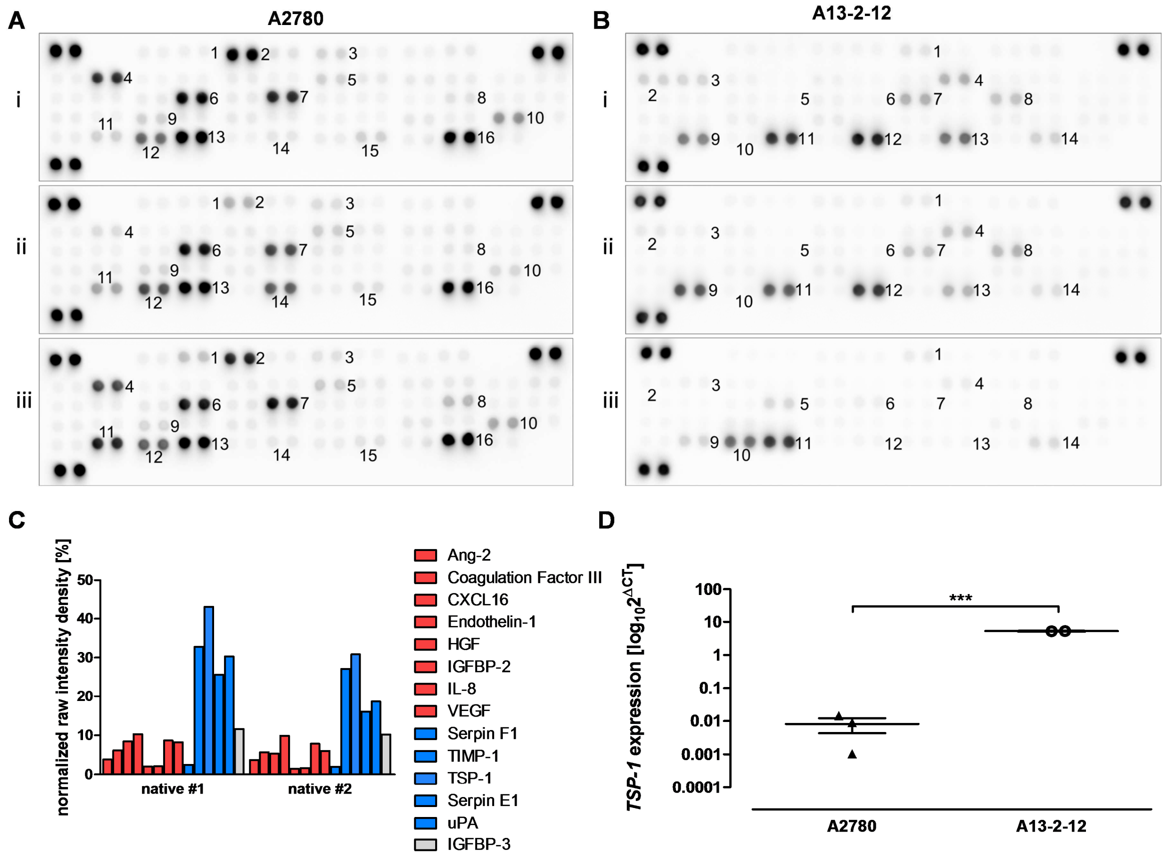

Suppl. Figure S9 Immunodetection of known regulators of angiogenesis using the Proteome Profiler™ Array. The highest signals are indicated respectively. **A** 72 h CM from A2780 **i** native, **ii** clone C7 *RUNX3* variant 1, **iii** clone B9 *RUNX3* variant 2 cells. Numbered spots: 1 ADAMTS-1, 2 Angiogenin (ANG), 3 Angiopoietin-2 (Ang-2), 4 CXCL16, 5 Endostatin, 6 HGF, 7 IGFβP-2, 8 TGFβ1 (LAP), 9 MMP-9, 10 PIGF, 11 Serpin E1, 12 Serpin F1, 13 TIMP-1, 14 Thrompospondin-1 (TSP-1), 15 uPA, 16 VEGF. **B** 24h CM from A13-2-12 **i** native, **ii** *RUNX3* variant 1, **iii** *RUNX3* variant 2 cells. Numbered spots: 1 Ang-2, 2 coagulation factor III, 3 CXCL16, 4 Endothelin-1, 5 HGF, 6 IGFβP-2, 7 IGFβP-3, 8 IL-8, 9 Serpin E1, 10 Serpin F1, 11 TIMP1, 12 TSP-1, 13 uPA, 14 VEGF. **C** Quantification of CM derived from native A13-2-12 cells generated from two different CM aliquots in independent experiments. No difference in the regulators could be detected. **D** Comparison of *TSP-1* expression in A2780 and A13-2-12 cells. * $p < 0.05$, ** $p < 0.01$, *** $p < 0.001$ in student t-test.
